# Modulation of nucleosomal DNA accessibility via charge-altering post-translational modifications in histone core

**DOI:** 10.1186/s13072-018-0181-5

**Published:** 2018-03-16

**Authors:** Andrew T. Fenley, Ramu Anandakrishnan, Yared H. Kidane, Alexey V. Onufriev

**Affiliations:** 1 Department of Physics, Virginia Tech, 2160C Torgersen Hall, Blacksburg, VA 24061 USA; 2 Genetics, Bioinformatics and Computational Biology Program, Virginia Tech, Blacksburg, VA 24061 USA; 3 Department of Computer Science, Virginia Tech, Blacksburg, VA 24061 USA; 4 Edward Via College of Osteopathic Medicine, Blacksburg, VA 24060 USA; 5 Center for Soft Matter and Biological Physics, Virginia Tech, Blacksburg, VA 24061 USA

## Abstract

**Background:**

Controlled modulation of nucleosomal DNA accessibility via post-translational modifications (PTM) is a critical component to many cellular functions. Charge-altering PTMs in the globular histone core—including acetylation, phosphorylation, crotonylation, propionylation, butyrylation, formylation, and citrullination—can alter the strong electrostatic interactions between the oppositely charged nucleosomal DNA and the histone proteins and thus modulate accessibility of the nucleosomal DNA, affecting processes that depend on access to the genetic information, such as transcription. However, direct experimental investigation of the effects of these PTMs is very difficult. Theoretical models can rationalize existing observations, suggest working hypotheses for future experiments, and provide a unifying framework for connecting PTMs with the observed effects.

**Results:**

A physics-based framework is proposed that predicts the effect of charge-altering PTMs in the histone core, quantitatively for several types of lysine charge-neutralizing PTMs including acetylation, and qualitatively for all phosphorylations, on the nucleosome stability and subsequent changes in DNA accessibility, making a connection to resulting biological phenotypes. The framework takes into account multiple partially assembled states of the nucleosome at the atomic resolution. The framework is validated against experimentally known nucleosome stability changes due to the acetylation of specific lysines, and their effect on transcription. The predicted effect of charge-altering PTMs on DNA accessibility can vary dramatically, from virtually none to a strong, region-dependent increase in accessibility of the nucleosomal DNA; in some cases, e.g., H4K44, H2AK75, and H2BK57, the effect is significantly stronger than that of the extensively studied acetylation sites such H3K56, H3K115 or H3K122. Proximity to the DNA is suggestive of the strength of the PTM effect, but there are many exceptions. For the vast majority of charge-altering PTMs, the predicted increase in the DNA accessibility should be large enough to result in a measurable modulation of transcription. However, a few possible PTMs, such as acetylation of H4K77, counterintuitively decrease the DNA accessibility, suggestive of the repressed chromatin. A structural explanation for the phenomenon is provided. For the majority of charge-altering PTMs, the effect on DNA accessibility is simply additive (noncooperative), but there are exceptions, e.g., simultaneous acetylation of H4K79 and H3K122, where the combined effect is amplified. The amplification is a direct consequence of the nucleosome–DNA complex having more than two structural states. The effect of individual PTMs is classified based on changes in the accessibility of various regions throughout the nucleosomal DNA. The PTM’s resulting imprint on the DNA accessibility, “PTMprint,” is used to predict effects of many yet unexplored PTMs. For example, acetylation of H4K44 yields a PTMprint similar to the PTMprint of H3K56, and thus acetylation of H4K44 is predicted to lead to a wide range of strong biological effects.

**Conclusion:**

Charge-altering post-translational modifications in the relatively unexplored globular histone core may provide a precision mechanism for controlling accessibility to the nucleosomal DNA.

**Electronic supplementary material:**

The online version of this article (10.1186/s13072-018-0181-5) contains supplementary material, which is available to authorized users.

## Background

Since the discovery of the structure of DNA [1] and the pioneering research on the structure of chromatin [2–4], the hunt has been on for trying to solve exactly the mechanisms which allow eukaryotic cells to manipulate access to any given region of their DNA as the first step in gene regulation. Elucidation of such mechanisms within eukaryotic cells is complicated by the sheer difference in length scales between the nucleus, about one micron in diameter, and the DNA stored inside, which can exceed a meter in length depending on the organism [5, 6]. Eukaryotic cells achieve the necessary amount of DNA compaction to fit within the nucleus via multiple levels of structural organization. Physical interactions [7–9] underpinning the various levels of this organization yield clues into the mechanisms behind the retrieval of genetic information in such a condensed environment, which are critical to the cell’s viability.

The first, and arguably the most fundamental, level of the chromatin structural organization is the nucleosome, where about 150 base pairs of the highly negatively charged DNA repeatedly wrap around a positively charged disk-like protein core consisting of two copies of the four histone proteins H2A, H2B, H3, and H4 [10–14]. Understanding how the cell controls accessibility to DNA sterically occluded within a nucleosome is crucial for gaining insight into the mechanism of gene regulation. A key question is how does a cell isolates and marks particular nucleosomes containing transcription sites for genes that are critical for maintaining a certain cell type and/or necessary for the cell to respond to environmental stress? One specific mechanism, supported by mounting experimental evidence, is that cells utilize reversible structural modifications to the histone proteins such as acetylation, methylation, ubiquitination, crotonylation, or phosphorylation, specific to certain amino acids within the histone proteins [15–19].

These post-translational modifications (PTMs) are capable of causing a wide range of structural and biological responses within the chromatin, including regulation of gene expression and silencing, DNA damage control, and chromatin rearrangement into heterochromatin [20–23]. Depending on the modification state of the PTM sites, they can act as markers for the binding of transcriptional factors [24] as well as directly modulate the strength of the interactions between the histone octamer and nucleosomal DNA [23, 25, 26]. Many of these PTM sites are located on the N- and C-terminus histone tails, which have been studied extensively, including atomistic modeling and simulation approaches [27–31]. PTMs located in the histone tails are generally not found to significantly contribute to the nucleosome core particle stability at physiological conditions [25, 32–34]; these PTMs are involved primarily in internucleosome interactions [7, 35], impacting higher-order chromatin structures [36, 37].

However, a potentially even larger number of biologically relevant sites capable of post-translational modification (PTM) are located within the globular histone core. These sites can directly and significantly impact the strength of DNA–histone association [25] and are expected to affect DNA accessibility [14, 38, 39]. Growing evidence, both theoretical [25] and experimental [40–42], suggests that charge-altering PTMs (acetylation, phosphorylation, crotonylation, propionylation, butyrylation, formylation, and citrullination) within the globular core of the nucleosome can have a significant and selective effect on accessibility of nucleosomal DNA by altering the strength of the DNA–histone association [41, 43].

At this point, we make a distinction between charge-altering PTMs such as acetylation, and those PTMs that do not affect the charge of the altered structure, such as methylation [44]. While both types of PTMs are of paramount biological importance, the distinction makes sense from a physics standpoint: electrostatics is the strongest force at the inter-atomic scale, and chromatin components at this scale are highly charged and strongly interacting, suggesting that predictive models [25, 45] of PTM effects may have to be different depending on the formal charge nature of the PTM considered. Throughout this work, PTMs are implied to be charge-altering unless otherwise stated.

Given the fundamental role of PTMs in epigenetics—in the control of DNA accessibility—and the sheer number of combined possible and known charge-altering PTMs in the histone core, we argue that the time is ripe for a general framework that offers a quantitative, causal connection between core histone PTMs and their effects on the nucleosomal DNA accessibility. Such a framework would allow one to rationalize in vitro experiments and formulate reasonable working hypotheses for the difficult in vivo studies aimed at investigating the potential biological impact of each PTM. As the amount of diverse data on PTMs grows rapidly, the absence of a unifying general framework that describes their effect on the same footing can hinder progress toward development of a detailed, mechanistic understanding of the key function of the nucleosome as the fundamental unit that controls accessibility of genomic information. In fact, close to seventy PTM sites located in the core and linker histones were recently detected [46], and these represent just a small subset of potential PTM sites not yet discovered; many sites are capable of supporting multiple types of PTMs. Only a tiny fraction of these potentially biologically relevant sites have been studied in detail [43, 47–49] and characterized in vivo [50], leaving much of the globular histone core’s role on modulating nucleosomal DNA accessibility still a mystery. Those very few core PTMs that have been explored in detail experimentally are almost exclusively the more familiar acetylation or phosphorylation, leaving the effect of many newly discovered types of PTMs, e.g., crotonylation, propionylation, butyrylation, formylation, and citrullination virtually unexplored in the context of globular core histones. At the same time, direct in vivo studies of charge-altering PTMs are very difficult; indirect genetic mimics such as K → Q mutation to mimic lysine acetylation are often used instead [51]. However, the approach has limitations: the effect of some of these mimics on chromatin compaction [52] and its function [53] are very different [54] from the original PTM they attempt to represent, and it is not known why.

The accessibility of the DNA in the nucleosome can be quantitatively characterized by the probability of a given DNA fragment to be in contact with the histone proteins, and thus occluded—the approach we are taking in this work. These probabilities are uniquely related to the key thermodynamic quantities of the DNA–histone complex: various free energy values that characterize the stability of the complex against partial to complete dissociation of its DNA and individual histones [14, 55]. For example, the free energy, Δ*G*, of stripping the DNA completely from the histone core is a measure for the overall stability of the histone–DNA complex and is estimated to be over 20 kcal/mol [25, 56], which is much larger than the stability of a typical protein [57, 58]. This very high stability of the nucleosome contributes to the key puzzle of its structure–function relationship: how can it be so stable while simultaneously providing access to the DNA when needed by the cell?

The effect of a given PTM on the DNA accessibility is likely to be more complex than simple partial unpeeling of the DNA from the intact histone core, since partially assembled states of the nucleosome, which can expose large regions of the DNA through removal of some of the core histones, are known to exist and to be important [14]. Some of these partially assembled states, such as the tetrasome—the complex of (H3–H4)_2_ tetramer with the DNA—are thought to be obligatory intermediate structures on the nucleosome assembly/disassembly pathway [22, 59, 60]. Furthermore, transient states in which the DNA is partially unwrapped off the lateral surface of the octasome [38] may be critical for facilitating transcription within an intact nucleosome [61]. Given that the various partially assembled states and transient states of the nucleosome all have varying degrees of DNA accessibility [62], the associated changes in their relative populations after a PTM is applied should be taken into account.

In what follows, we introduce a multistate, thermodynamic model that takes into account key partially assembled states of the nucleosome to study the potentially most impactful (charge-altering) PTMs within the histone globular core, i.e., acetylation and phosphorylation, on the strength of histone–DNA association and nucleosomal DNA’s accessibility. And we extend our analysis of charge-altered lysine residues to crotonylation [46], propionylation [63], butyrylation [63], formylation [64], succinylation [65], and hydroxyisobutyrylation [66].

## Results

Here we quantitatively characterize accessibility of the DNA in the nucleosome by the probability of the given DNA fragment to be in contact with the histone proteins (and thus protected from access by nuclear factors). We relate the accessibility patterns to biological effects such as transcription up- or down-regulation. We have two types of results: (1) the framework and (2) its predictions. Below is an outline of “Results” section intended to orient the reader.

We begin with a brief description of the proposed multistate framework (model) and its capabilities (technical details of the model are fully described in “Methods” section and in the Additional file 1). We proceed with the initial validation of the model against available thermodynamics data on the effects of core histone acetylation on nucleosome stability. We then describe how localization of the effect of a given PTM on the DNA accessibility is predicted within our framework. To enable a detailed, region-specific analysis of the PTM’s effect on nucleosomal DNA accessibility, we introduce the concept of a *PTMprint*—the distinct pattern of DNA accessibility resulting from an applied PTM. We show how PTMprints may be used to relate the anticipated biological impact with the predicted DNA accessibility change.

Next, we describe the many predictions of the framework, starting with the more general trends and then moving to the effects of several groups of PTMs and then down to individual PTMs. Using our multistate model, we are able to predict differences in DNA accessibility for nearly one hundred potential PTM sites within the globular histone core, i.e., the histone proteins sans their C- and N-terminus tails that could have a charge modifying PTM. Multiple connections to experiment are made; connections to thermodynamic measurements are mostly presented in “Results” section, while “Discussion” section includes the proposed connection to biological effects, with references to Additional file 1 for more details where appropriate.

### Outline of the theoretical framework

**Fig. 1.**
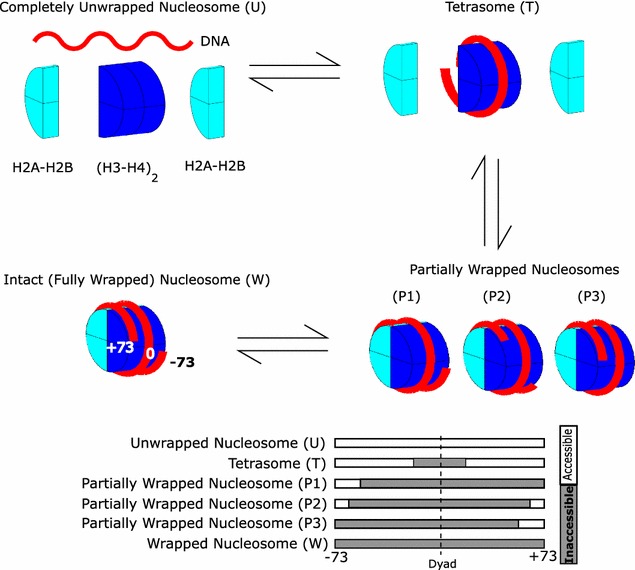
The six conformational states used in the thermodynamic model of DNA accessibility in the nucleosome. The states differ by the degree of DNA accessibility and histone core composition. The DNA is completely inaccessible in the canonical nucleosome (W, wrapped). In each of the three partially wrapped conformations (P1, P3, P3) 20 bp are accessible. In the tetrasome (T), a total of 69 bp are accessible [62]. Each conformational state can also carry a post-translational modification (PTM) resulting in a total of twelve distinct energy states considered

Our physics-based framework (model) predicts the change in accessibility of different regions of the wrapped nucleosomal DNA in response to charge-altering PTMs such as lysine acetylation within the globular histone core. To enhance biological realism of our physical model, we account for key known transition states along the primary nucleosome assembly/disassembly pathway [14, 38, 41, 67], each of which exposes different regions of the nucleosomal DNA, see Fig. 1. As we will demonstrate, the multistate nature of the model is essential for making several counterintuitive predictions.

The model, based on classical atomistic electrostatics and general thermodynamics, accounts for the modulation of accessibility of the different regions of nucleosomal DNA by calculating how a given charge-altering PTM alters electrostatic interactions within the nucleosome, thereby shifting the relative energetics of the represented nucleosome states. Specifically, the free energy of each state relative to the free energies of all of the other states effectively determines the probability *P* that the system will adopt a particular state and its associated DNA accessibility, see "Methods" section. Depending on the location of the applied PTM, the relative free energies of the different states can change appreciably, and thus significantly alter the probabilities of occurrence for each state. By comparing the calculated probabilities P* of the states post-PTM, with the probabilities *P* pre-PTM, our model can quantitatively estimate the relative change in probabilities P*/*P* (accessibility) of the corresponding DNA regions, which is the key quantitative outcome of the model. For example, a tenfold increase in accessibility of a given state of the nucleosome upon application of a PTM means that the system is now in this state ten times more often than before the PTM was applied.

### Predicted PTM-induced changes in DNA affinity to the nucleosome core are in agreement with known experimental values

To validate our multistate model, we have compared the predicted destabilization of the histone–DNA complex upon the application of the few PTMs with known experimental values, Table 1. The predicted values are within the experimental error margins for these three PTMs, which is strong evidence in support of our purely physics-based model that has no fitting parameters.

**Table 1:** Predicted destabilization of the nucleosome, ∆∆*G*, due to histone acetylation compared to available experimental values as quantitative validation of the model [41, 43]

Acetylated group	Experimental ΔΔ*G* (kcal/mol)	Predicted ΔΔ*G* (kcal/mol)
H3K56	2.0 ± 0.6	2.3 ± 0.4
H3K115	0.4 ± 0.2	0.2 ± 0.01
H3K122	0.2 ± 0.2	0.3 ± 0.01

The experimental uncertainty is discussed in the Additional file 1; errors in the predicted values are discussed in “Methods” section

The available experimental values in Table 1 are all with regard to acetylation of lysines. For reasons we discuss in “Methods” section, among various types of PTMs, the model is expected to be most accurate for those charge-altering PTMs, such as acetylation, that entail small structural changes to the modified residue. Therefore, given the quantitative agreement with available experimental data regarding acetylation, our main focus here is the results on lysine acetylation within the globular histone core. We also provide a discussion regarding other PTMs associated with similar small structural changes, i.e., crotonylation, propionylation, butyrylation, formylation, succinylation, and hydroxyisobutyrylation. The model is capable of mimicking the effect of phosphorylation, which is also a charge-altering PTM; however, for reasons discussed in “Methods” section, these results are more qualitative.

### Accounting for localization of the impact on the DNA accessibility per charge-altering PTM in the histone core

**Fig. 2:**
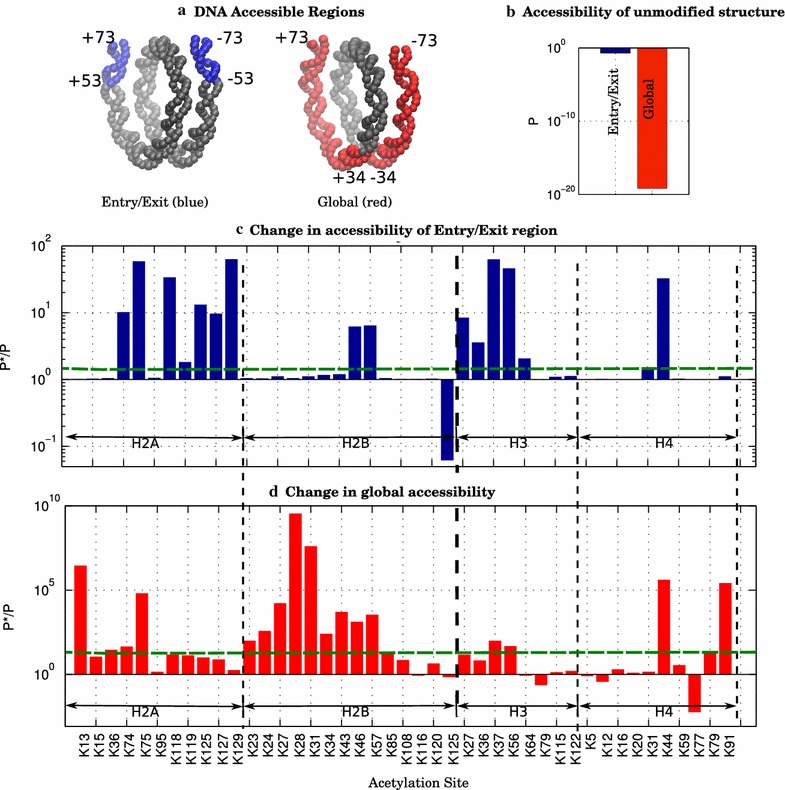
Nucleosomal DNA accessibility PTMprints for acetylated lysine residues within the globular histone core. **a** Schematic of the different DNA regions. **b** Accessibility of the different nucleosomal DNA regions without a PTM. The very low probability of the global region accessibility corresponds to the simultaneously unwrapping of 78 bp of the DNA. **c** and **d** show change in accessibility, (P*/P), upon lysine acetylation, by region. **c** Entry/Exit, and **d** global region. The horizontal dashed lines indicate the conservative threshold value used to define a functionally significant change in accessibility. The threshold value corresponds to the accessibility change of H4K31 for the entry/exit region, and to H4K79 for global accessibility. Note that the entry/exit nucleosomal DNA is accessible in multiple states (U, T, P1, P2, P3); the global region is only accessible in the T and U states, Fig. 1

Our model is able to determine PTM-induced changes in accessibility for two major regions of nucleosomal DNA, which we refer to as the *entry/exit* and *global* regions (Fig. 2a). The entry/exit region, which refers to the 10 bp at the 3’ or 5′ ends of the DNA near the dyad, is potentially accessible via natural exposure of the transiently unpeeling DNA ends. PTMs classified as entry/exit change the accessibility of just this small region without necessarily increasing accessibility of the rest of the DNA. However, PTMs classified as global, which contain the entry/exit region, increase accessibility of most of the DNA simultaneously. It is also important to recognize that the native (no PTMs) accessibility of these two regions for the intact nucleosome is dramatically different, Fig. 2b. And so, a relatively small change in the entry/exit probability is potentially more significant than a relatively larger change in the global region. We find that certain PTMs can greatly enhance the rate of the DNA exposure without resulting in complete dissociation of the nucleosomal DNA or decomposition of the histone core. The global region allows for a significantly greater exposure of the nucleosomal DNA, about halfway (40 bp) to the dyad, and therefore only becomes accessible after a considerable level of destabilization to the nucleosome.

For any acetylated lysine within the histone globular core, Fig. 2c, d shows the associated change in accessibility of the two regions of nucleosomal DNA. Importantly, not all PTMs that significantly change accessibility of one region of nucleosomal DNA simultaneously change accessibility of the other region. The model shows that the specific location of the PTM in the nucleosome is important, thus providing a more nuanced approach to the modulation of the DNA accessibility in a region-specific manner. The very important consequences of this finding will be explored further below.

### PTMprints

The ΔΔ*G* value of any applied PTM does not immediately convey the change in DNA accessibility nor does the value itself hint at the subsequent potential modulation in transcription rates caused by the PTM. To better interpret the biological consequences of the applied PTMs, we first group the PTMs based on their associated DNA accessibility profile, Fig. 2c, d, using the following classification scheme: (1) the entry/exit region only changes (2) the global region changes, (3) both entry/exit and global region change, and (4) weak changes to DNA accessibility. Once grouped based on classification, those PTMs within a group that have known biological effects are then used as a guide to infer potential effects of the other, as yet investigated, PTMs within the same group.

The threshold accessibility values between the classifications are determined based on globular core PTMs whose biological role has been previously investigated experimentally [50], Table 2. We have set the 1.5-fold change in accessibility corresponding to H4K31^Ac^ as our threshold value for biological significance for the entry/exit region. Critically, the seemingly low 1.5-fold change in nucleosomal DNA accessibility has been shown to have direct in vivo consequences (see below), including an order of magnitude increase in steady-state transcript levels [68] and promoter activity [69]. For the global region, the significance threshold of 19-fold corresponds to H4K79^Ac^. These PTMs, according to Fig. 2c, d, are “exclusive” to their respective regions: H4K31^Ac^ modulates entry/exit accessibility only, while H4K79^Ac^ is the only core PTM with the lowest predicted fold change that just affects the global region accessibility. The model also identifies PTMs that show increases in accessibility for both regions of the DNA, with H4K44^Ac^ showing the greatest amount of simultaneous accessibility change. Finally, weak accessibility changes are attributed to any PTM that falls below the thresholds defined above. Note that those PTMs that are classified as having a weak effect on DNA accessibility may still be biologically significant for at least two reasons: (1) Our 19-fold threshold corresponding to H4K79^Ac^ may be too conservative. (2) The “weak” PTMs could serve as transcription factor markers and thus indirectly facilitate DNA accessibility [70].

**Table 2:** PTMprint classification for all possible acetylations per core histone

Classification/histone	H2A	H2B	H3	H4
Change DNA accessibility in entry/exit	K118, K119, K127, K129	K125*	K36, K64	K31
Change DNA accessibility globally	K13, K15, K36	K23, K24, K27, K28, K31, K34, K43, K85		K79, K91, H4K77*
Change DNA accessibility in both regions	K74, K75, K125	K46, K57	K27, K37, K56	K44
Weak effect on DNA accessibility	K95	K108, K116, K120	K79, K115, K122	K5, K12, K16, K20, K59

PTMs predicted to have non-weak *stabilizing* effect on the nucleosome, and thus a *decrease* in the DNA accessibility, are marked by asterisk

To enable a detailed, region-specific analysis of the PTM’s effect on nucleosomal DNA accessibility, we introduce the concept of *PTMprint*, Fig. 2c, d, which indicates the location and predicted strength of each PTM. Thus, each applied PTM results in its own DNA accessibility profile or PTMprint. The biological implications of this concept in the context of known PTMs and our predictions are presented in “Discussion” section. Table 2 provides the predicted PTMprint classification for all acetylations per each core histone.

### Correlation between PTM location and its effect on DNA accessibility

**Fig. 3:**
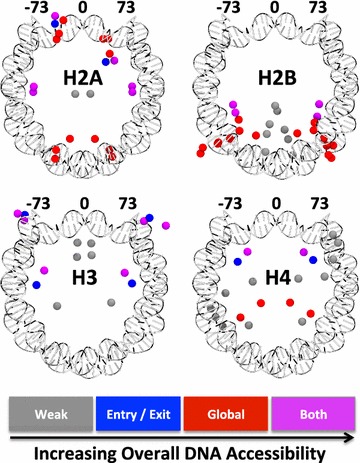
The spatial distribution of residues susceptible to acetylation throughout the globular core for each histone protein. Each PTM for a given histone is color coded according to its PTMprint classification: its predicted change on accessibility of different regions of the DNA

Each set of potential acetylation sites (including many sites not yet characterized in vivo) within a particular histone occupies unique regions throughout the octamer relative to the location of a given segment of the nucleosomal DNA, Fig. 3. Additionally, due to the nature of the wrapped DNA, certain segments of the DNA can be in close spatial proximity in 3D space, but distant in the sequence. Perhaps the most prominent example is the dyad, which is spatially near both the 3′ and 5′ ends of the DNA yet maximally distant in sequence from either end. This contrast of “near in physical distance” while “far in sequence distance” at the dyad leads to many acetylation sites in the region that increase the breathing fluctuations of the 3′ and 5′ ends while only moderately increasing the accessibility of the global region, see Fig. 3. Furthermore, while all histone proteins except H2B contain potential acetylation sites near the dyad, H2B contains potential acetylation sites predicted here to increase the accessibility of the global region by the greatest amount relative to all other sites throughout the octamer.

A natural question arises: Is there a simple correlation between a PTM location and its effect on the DNA accessibility? A general trend is already clear from Fig. 3: PTM sites with a strong effect tend to be near the DNA. The trend is intuitive, but by no means strongly predictive: there are many exceptions. For example, H4K91 is far from the DNA, and yet its acetylation is predicted to globally increase DNA accessibility. Conversely, many sites in close proximity to the DNA are predicted (and some already found experimentally, e.g., H3K115, H3K122) to have a weak effect on the DNA accessibility, see the Additional file 1 for a detailed analysis. In summary, the effect of a given PTM on the nucleosomal DNA accessibility stems from a complex interplay of non-trivial electrostatic interactions and multiple states of the nucleosome and cannot be predicted faithfully from seemingly simple and intuitive PTM location metrics. In other words, there are no simple proxies for rigorous thermodynamic quantities such as ΔΔ*G* or P*/*P* to quantify the effect of each PTM on the DNA accessibility. Ultimately, we would like to infer about the biological impact of a given PTM based on its predicted effect on the DNA accessibility: Our approach to this challenge is presented in “Discussion” section; it is based on the PTMprint concept.

### Additive effect of multiple simultaneous PTMs

The nucleosome can contain multiple simultaneous PTMs applied to various residues throughout the histone octamer—Is their combined effect additive or cooperative? Specifically, when considering the impact of multiple simultaneous PTMs, the net change in free energy difference between accessible and inaccessible states, ΔΔ*G*^1+2^, is not necessarily equal to the sum ΔΔ*G*^1^ + ΔΔ*G*^2^. To our knowledge, there is only one case of experimentally determined ΔΔ*G* for a pair of acetylation PTMs: H3K115^Ac^ and H3K122^Ac^ [43]. As shown in Table 3, in this particular case the nonadditivity is negligible, consistent with our calculated values within the margin of experimental error. However, no statistically meaningful conclusions can be drawn from a single case (A recent experimental data point that mixes two types of PTMs, H3Y41^Ph^/H3K56^Ac^, also shows additivity within experimental error [71]).

**Table 3:** Additivity of multiple PTMs

	ΔΔ*G* (kcal/mol) for the acetylation of			Difference
	H3K115	H3K122	H3K115 and H3K122	
Experimental data	0.4 ± 0.2	0.2 ± 0.2	0.6 ± 0.2	0.0 ± 0.35
Calculated values	0.2 ± 0.01	0.3 ± 0.01	0.3 ± .0.01	0.2 ± 0.01

Comparison of experimentally determined and calculated ∆∆*G* for a PTM pair

**Fig. 4:**
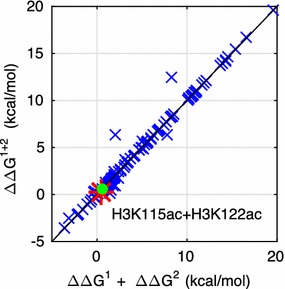
Additivity of the effect of multiple PTMs. Shown are the relative changes in free energy for different combinations of PTMs (∆∆*G*^1+2^) compared to the sum of individual PTMs (∆∆*G*^1^ + ∆∆*G*^2^). In rare cases (two outlier blue crosses), the change in ∆∆G^1+2^ due to a pair of PTMs can be significantly different from the sum of the changes in binding affinity for each of the PTMs separately (∆∆*G*^1^ + ∆∆*G*^2^). The green dot identifies the PTM pair for which experimental data are available, [43], and the red star shows the corresponding predicted value

To make a statistically significant conclusion about possible PTM additivity and understand the origin of nonadditivity, here we have considered 113 random pairs of acetylated lysines, for which we calculate ΔΔ*G*^1+2^ versus ΔΔ*G*^1^ + ΔΔ*G*^2^, Fig. 4. The vast majority of cases show nearly perfect additivity between a pair of PTMs, with RMSD of 0.66 kcal/mol from perfect additivity, which can be treated as zero within the model’s accuracy. The additive effect of multiple PTMs on ΔΔ*G* is the equivalent of their multiplicative effect on DNA accessibility (expressed as probability). Although ΔΔ*G* in Fig. 4 corresponds to global DNA accessibility, we find (results not shown) that the nearly perfect multiplicative effect also applies to the entry/exit region accessibility.

However, within the set of randomly selected pairs of PTMs we have found two noteworthy exceptions of the “majority additive” rule: cases with significant (> 3 kcal/mol) nonadditivity. The simultaneous acetylation of H4K79 and H4K44 results in ΔΔ*G*^1+2^ = 13.5 kcal/mol compared to 1.7 + 7.7 = 9.4 kcal/mol—the sum of the ΔΔ*G* for each of the two sites separately, and the simultaneous acetylation of H4K79 and H3K122 results ΔΔ*G*^1+2^ = 6.4 cal/mol compared to 1.7 + 0.2 = 2.0 kcal/mol—the sum of the ΔΔ*G* for each of the two sites separately. We show, see the Additional file 1, that nonadditivity between pairwise PTMs is a direct consequence of the nucleosome–DNA complex having more than two states, Fig. 1.

### Other charge modifying PTMs of lysine

**Fig. 5:**
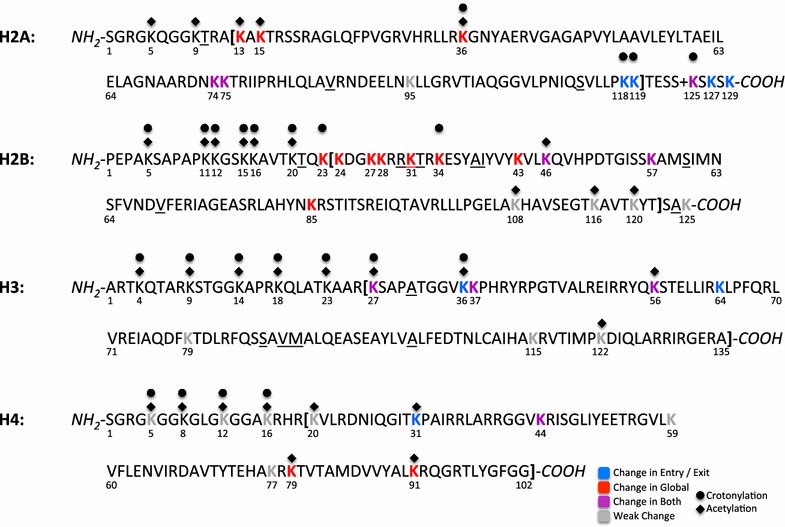
Histone sequences with all lysine residues annotated with experimentally known PTMs and color coded by our predicted effect on the DNA accessibility as in Figs. 4 and 5. Regions within the bold square brackets [] are considered part of the globular core, which is the focus of this work. Underlined residues denote mutations relative to the human variant of the histone. The ‘+’ in H2A refers to a missing residue relative to the human variant of H2A. Note that in the nucleosome crystal structure used here (PDB ID 1KX5) H4 is the only human histone variant; H2A and H2B are from *xenopus* and H3 is a bovine variant

Modifying the charge of lysine appears to be a common mechanism in nature to modulate accessibility of nucleosomal DNA. There are many other recently discovered PTMs that neutralize the charge of lysine in an analogous way as acetylation: crotonylation [46], propionylation [63], butyrylation [63], formylation [64], succinylation [65], and hydroxyisobutyrylation [66]). Significantly, some of these charge-altering modifications can target additional lysines, e.g., H2AK118, in the histone core than have previously been found acetylated, see Fig. 5.

### Crotonylation, propionylation, butyrylation, and formylation

Our model applies to crotonylation, propionylation, butyrylation, and formylation of lysine at a similar quantitative level as the acetylation of lysine, see “Methods.” For example, the model predicts that the acetylation PTMprints shown in Fig. 2 would apply to any lysine with one of these modifications in Fig. 5, albeit semiquantitatively for crotonylation and butyrylation, see “Methods” section. The biological effects of these four types of PTMs should, by and large, be similar to those predicted for acetylation, see “Discussion” section.

There are some known crotonylation sites whose status with respect to acetylation remains to be investigated experimentally: H2AK118, H2AK119, H2AK125, H2BK23, and H2BK34 [46], see Fig. 5. Our model predicts the overall effect on histone–DNA association (ΔΔ*G*) of these sites (either acetylated or crotonylated) as: 0.5, 1.5, 0.7, 2.7, and 3.3 kcal/mol, respectively. Most of these changes are not negligible, comparable to the effect of H3K56^Ac^ on the nucleosome–DNA association. The model predicts H2AK118^Cr^ and H2AK119^Cr^ to increase DNA accessibility in the entry/exit region and H2AK125^Cr^, H2BK23^Cr^, and H2BK34^Cr^ to increase DNA accessibility in the global region, just like in Additional file 1: Table S4.

### Hydroxyisobutyrylation and succinylation

Hydroxyisobutyrylation replaces the hydrogens from the methyl group with two methyl groups and a hydroxyl group. Given the extra polar interactions, the hydroxyl could make, and the potential steric clashes of the additional methyls, we suggest the results of our model for acetylation can be extended to hydroxyisobutyrylation only very qualitatively. Similar reasoning applies to succinylation, which extends the methyl group with an acetic acid group.

### Charge-altering PTMs of residues other than lysine

Modifying lysine is not the only strategy nature can adopt to modulate the charge of the histone core to facilitate access to particular regions of nucleosomal DNA. Below we consider these charge-altering PTMs in some detail.

### Phosphorylation

Phosphorylation [23, 47, 49, 71] of normally neutral residues (i.e., serine, threonine, and tyrosine) results in a negative net change of the phosphorylated residue, which, from the general physical argument [25] can be expected to also have a disruptive effect on the DNA–core affinity as histone acetylation.

Phosphorylation chemically modifies a different set of residues (serine, threonine, and tyrosine) than lysine modified by acetylation and other PTMs considered above. We predict that most of the candidate phosphorylation residues would substantially increase nucleosomal DNA accessibility in one (entry/exit or global) or both regions, Additional file 1: Figure S1. Generally, the destabilizing effect of most phosphorylations on the nucleosome is strong, and the strength is expected: The residues that are subject to phosphorylation in the nucleosome core are mostly buried inside the low dielectric core, changing their charge from 0 to -1 is energetically costly. The largest predicted increase in DNA accessibility comes from phosphorylations of H4Y98, H4T80, H4Y72, H3T118, H3T58, nearly all possible phosphorylations of H2B, and that of H2AT76, and H2AT101. In the case of phosphorylation, we refrain from making detailed quantitative conclusions for reasons discussed in “Methods". Still, it is reassuring that the predicted global effect of H3T118^P^ and H4S47^P^, and their relatively large predicted ΔΔ*G* of 8.1 and 2.7 kcal/mol, respectively, are consistent with the experimental role of these PTMs as affecting global DNA accessibility [23]. On the other hand, H3Y41^P^, H3T45^P^ and H3S57^P^, implicated experimentally as affecting entry/exit accessibility, exhibit lower ΔΔ*G*: 0.4, 1.4, and 2.5 kcal/mol. According to our model, these PTMs have strong signature in the entry/exit PTMprint region, Additional file 1: Figure S1, but are only borderline “global,” quite likely within the error margin of the model.

### Citrullination

Citrullination is probably the only known charge-altering PTM that is not considered here. It converts arginine to citrulline via the replacement of the primary ketimine group with a ketone group that essentially neutralizes the charge. Within the histone protein, this conversion antagonizes arginine methylation and might not be reversible [72–75]. Given that many histone arginines play an important role via interacting with the minor grooves of the wrapped DNA [76, 77], citrullination of arginines within the histone core could have a significant, non-reversible, impact on nucleosome stability. In the future, this PTM should be straightforward to model due to the sterically small chemical modification resulting in a localized change in charge.

## Discussion

### PTMprint: connection between PTM strength, location, and biological impact

Having quantified the effect of every possible lysine acetylation within core histones on the DNA accessibility in the nucleosome, we can use the resulting PTMprints to discuss possible connections with the PTM effects on the in vivo phenotypes. Given the complexity of in vivo networks that control key cellular processes, there is little hope of making such connections by purely theoretical reasoning. The very existence of such a connection depends critically on answers to the following two questions, which, at the moment, can only be addressed experimentally: (1) Do non-specific, e.g., independent of the particular DNA sequence, changes in the DNA accessibility matter in vivo? And if so, (2) what is the threshold of biological significance for the nucleosomal DNA accessibility change? Fortunately, a body of recent experimental work investigating a connection between the nucleosome stability [78] and gene expression [69, 79] has established that increases in nucleosomal DNA accessibility as small as 1.5-fold can have significant biological consequences, e.g., up to an order of magnitude increase in steady-state transcript levels [68] and promoter activity [69]. Importantly, these biological consequences of increased DNA accessibility are not sequence-specific, i.e., the effects are the function of the increased DNA accessibility per se.

Combining these two experimental facts with our quantitative results allows us to propose the following interpretative framework based on the computed changes in the nucleosomal DNA accessibility for each of the PTMs (e.g., Fig. 2 for acetylations). *We predict that if a certain biological effect directly depends on the DNA accessibility, then PTMs with similar PTMprints should, on average, have similar biological roles when applied in similar context. We stress the context aspect: PTMs that are similar by their accessibility PTMprints could still have dramatically different biological outcomes by the consequence of exposing different genes*. Likewise, loosening of the nucleosome structure by a charge-altering PTM could be utilized during the nucleosome assembly process, while the same PTM could also be used to fine-tune transcription at a different stage of cell cycle. However, even with these caveats, the suggested PTMprint similarity may be used as a reasonable starting hypothesis when investigating new PTMs in vivo, or known PTMs but in a different context. Below we demonstrate the approach on several concrete examples, including known PTMs and our predictions.

### Effects of PTMs in the globular histone core: predicted general trends

The absolute majority of histone acetylations are predicted to decrease the strength of histone core–DNA association, and thus increase DNA accessibility, Fig. 2, consistent with the expected effect of decreasing charge–charge interaction between the globular histone core and the DNA upon charge neutralization within the core [25]. Since the experimental threshold of the effect of altered DNA accessibility on transcription is very low, we thus predict that most charge-altering PTMs will up-regulate transcription, to highly variable degrees. The prediction is consistent with the measured effect of the handful of known PTMs [23, 48, 50], see Additional file 1: Table S4.

**Fig. 6:**
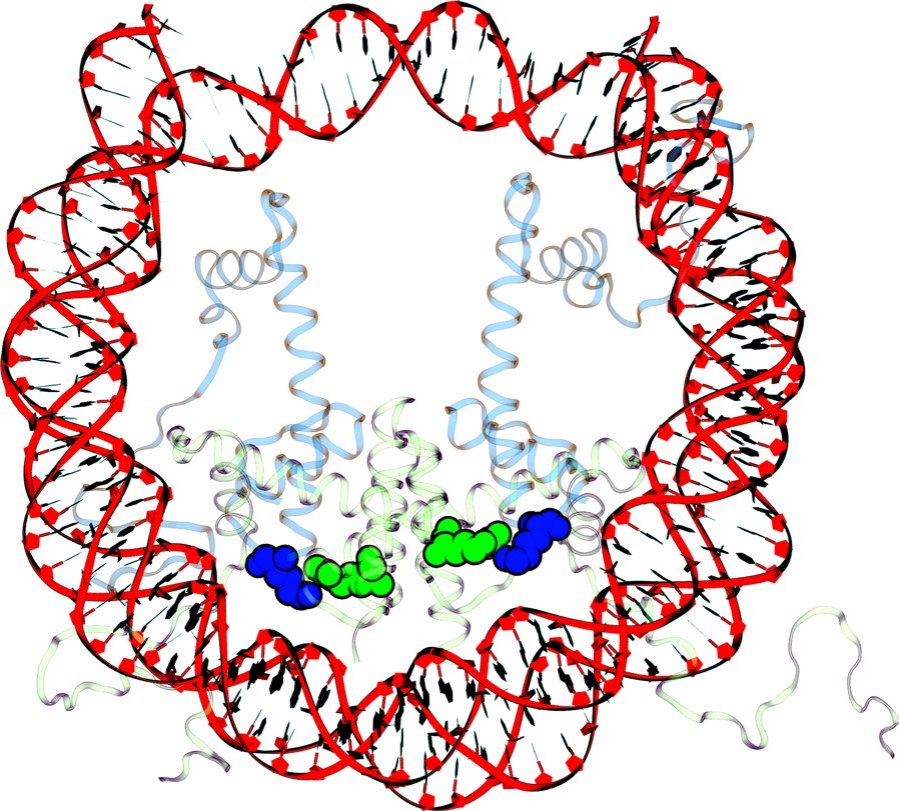
Explanation for the counterintuitive net stabilizing effect of H4K77^Ac^ on the nucleosome, which makes the global DNA less accessible. In the intact nucleosome, the positively charged H4K77 (blue spheres) is in close proximity of H2BR92 (green spheres): their repulsion destabilizes the nucleosome, favoring more open states with higher DNA accessibility. This destabilizing interaction is eliminated when an acetylation of H4K77 neutralizes its positive charge

However, completely counterintuitively, a few lysine acetylations, notably H4K77 and H2BK125, are predicted to result in the opposite effect—a decrease in the DNA accessibility, which could result in depressed transcription. In the case of H4K77, an experimental confirmation of the biological consequence of this counterintuitive trend is already available: It does correlate with a repressed chromatin phenotype [80]. We propose the following physics-based, structural explanation for the effect, Fig. 6, where we analyze the case of H4K77^Ac^ for which the counterintuitive trend is by far the strongest, Fig. 2 (also Additional file 1: Table S1). In the intact nucleosome, H4K77 is in close proximity, within 2.4 Å, of H2BR92, see Fig. 6. Since both residues are protonated, and thus positively charged, their close proximity is electrostatically highly unfavorable. In addition to the fully wrapped (intact) state of the nucleosome, the same close proximity and the repulsion between H4K77 and H2BR92 are “inherited” by several other partially assembled nucleosome states, namely the partially unwrapped and the tetrasome states, Fig. 1. This unfavorable interaction is, however, absent in the disassembled nucleosome, in our case the “unwrapped” state, Fig. 1. Thus, in the native nucleosome without PTMs, the H4K77–H2BR92 like-charge repulsion provides a certain amount of bias toward the fully disassembled nucleosome in which the DNA is unwrapped and fully exposed. An acetylation of H4K77 has two effects: just like any other charge-neutralizing PTM it abolishes some of the stabilizing opposite charge attraction between the histone and the DNA, promoting more open, unwrapped structures. But, unlike most other charge-neutralizing PTMs, acetylation of H4K77 also abolishes the strong unfavorable like-charge repulsion with H2BR92, thus favoring the fully wrapped and other “wrapped” states of the nucleosome including the tetrasome, Fig. 1. As our calculations for H4K77 demonstrate, the balance between these two opposite effects is such that second one wins over the first, resulting in a net decrease in the DNA accessibility for this unusual PTM.

Another important prediction, Figs. 2 and 3, is a significant heterogeneity of the magnitude of individual PTM effect on the DNA accessibility. Specifically, we find a plethora of lysines, e.g., H4K44, H2AK75, and H2BK57, that when acetylated, are predicted to increase the entry/exit or global accessibility of the nucleosomal DNA significantly more than the extensively studied acetylation sites within histone H3, i.e., H3K36, H3K56, H3K115, and H3K122 [41, 43, 81, 82]. In particular, histones H2A and H2B contain lysines (H2AK13, H2BK28, and H2BK31) that when acetylated are each predicted to cause over a million or even billion-fold change in accessibility of the global region relative to an unmodified nucleosome. Histone H4 also contains two relatively significant modification sites for increasing accessibility, the predicted H4K44 and known H4K91. It is important, however, to always consider these seemingly very large changes in the context of the very low accessibility of the global region in the intact nucleosome, Fig. 2b. Thus, the absolute probability for the corresponding DNA region to be exposed in the PTM state, P*, can remain small which is consistent with the general notion that the nucleosomal DNA remains well protected. Also, the global region is heterogeneous with respect to propensity to the DNA unwrapping [38, 39]: A global PTM site close to the entry/exit region can be made much more exposed in absolute terms by the same strength PTM than a site opposite of the dyad. For example, histone H3 contains no lysines whose acetylation is predicted to change global accessibility beyond one hundred-fold, yet many of the lysines within H3 have known biological impact when acetylated, consistent with the fairly low threshold of potential biological importance. Currently, our model only distinguishes between either 20 bp (entry/exit) or 80 bp (global) of DNA accessibility, it is possible that some PTMs currently classified as “weak” could provide access to DNA regions in-between the entry/exit and global boundaries, if the model were to be expanded to include a greater number of partially unwrapped states of the nucleosome.

A number of even more general observations can be made based on Fig. 2 and Table 2: (1) H2B is relatively rich in PTMs that have relatively strong global effect; (2) H2A is relatively rich in PTMs that predominantly affect accessibility of the entry/exit DNA, relatively strongly. (3) H2A and H3 have the most PTMs that have dual affect in both regions. (4) H2B is relatively rich in “weak” entry/exit PTMs; (5) the majority of H4 PTMs are weak, but with some very notable exceptions discussed above.

A number of core histone acetylations are predicted to result in little change in the DNA accessibility (weak), e.g., H2AK95^Ac^ with P*/*P* ≈ 1, which may indicate minimally disruptive PTMs used by the cell as markers, e.g., to initiate binding of a regulatory factor. For this reason, and due to accuracy limitations of the model, the predicted very weak direct impact on DNA accessibility should not be misinterpreted as a prediction of no biological consequence.

### PTMprint: specific examples

*H3K56*^*Ac*^ We begin with acetylation of H3K56, Additional file 1: Table S4, which is arguably the most “multi-functional” acetylation among those few charge-altering PTMs in the globular histone core that are already well-characterized in vivo. Experimental genome-wide evidence suggests that acetylation of H3K56 is necessary for efficient gene transcription [83] along with facilitating DNA replication and preventing epigenetic silencing [84, 85]. Our framework provides an intuitive explanation for this versatility: among acetylations already characterized in vivo, H3K56^Ac^ is the only one that leaves a significant PTMprint (increase in accessibility) in both the entry/exit and global regions, Fig. 3, which suggests why H3K56^Ac^ is “multi-functional.” Moreover, the predicted increases in nucleosomal DNA accessibility resulting from this PTM are well above the “significance” threshold of 1.5-fold change discussed above; the PTM can be used by the cell for fine-tuning (up-regulating) of the processes that need more accessible DNA via loosening of the nucleosome structure, e.g., transcription. Based on the PTMprint similarity to H3K56, we suggest that H3K37, H3K27 (and, possibly H3K36 which is just below our significance threshold for entry/exit) are worth investigating as well—they may turn out to be rich, multi-functional PTMs.

*H4K91*^*Ac*^ This is another noteworthy example—among experimentally characterized core acetylations, Additional file 1: Table S4, H4K91^Ac^ stands out as one of the strongest—10^5^ predicted increase in global accessibility of the nucleosomal DNA (Fig. 2d). The acetylation of H4K91 is one of the few, if not the only PTM so far known to regulate nucleosome stability through altering histone–histone interactions [50, 86]. It is reasonable to assume that large global changes in the accessibility will have a non-negligible biological effect (transcription up-regulation, partial octamer disassembly, etc.) Also, loosening of the DNA–nucleosome association may be necessary for correct assembly of the nucleosome [25]. Our model shows that the global region, see Fig. 2d, shows a marked increase in accessibility while the region corresponding to the 5′ and 3′ ends of the nucleosomal DNA shows almost no difference in accessibility, see Fig. 2c. This accessibility profile is a direct result of H4K91 being located at the H3-H4 tetramer-H2A-H2B dimer interface, and acetylation weakening the interactions between the dimers and tetramer such that the preference for the system to be in tetramer state, see Fig. 1, increases.

*H4K44*^*Ac*^ Next, we turn our attention to H4K44^Ac^, which belongs to the same PTMprint classification as H3K56^Ac^—a PTM that affects both the entry/exit and global accessibility. The geometric location of this lysine in the nucleosome core is similar to that of H3K56 (see Additional file 1), further suggesting that the two PTMs may have similarities. Moreover, H4K44^Ac^ predicted effect on DNA accessibility is larger than that of H3K56^Ac^, in both entry/exit and global regions. The PTM’s thermodynamic effect on the nucleosome stability is ΔΔ*G* = 7.7 kcal/mol, compared to 2.3 kcal/mol of H3K56^Ac^. In fact, in the global region H4K44^Ac^ increases DNA accessibility by 100,000-fold, as much as H4K91^Ac^ (Fig. 2d), which is among the acetylations that have the strongest effect. In summary, we predict that H4K44^Ac^ will have a noticeable biological effect as well, and that this effect might bear resemblance to the effects H3K56^Ac^, and, to some extent, H4K91^Ac^. At the same time, we do not exclude the possibility that biological effect of H4K44^Ac^ may be too strong, possibly even lethal in some cases [20].

*H3K115*^*Ac*^ and *H3K122*^*Ac*^ These two previously studied acetylations are classified here as having a “weak” effect on DNA accessibility, consistent with their relatively low predicted/measured ΔΔ*G*, and at most 1.4- to 1.6-fold change in DNA accessibility upon acetylation, Table 2. Counterintuitively, both sites are close to the DNA, especially H3K122, so why is the effect so weak? Our explanation for the weak effect of these PTMs is that they are physically close to the entry/exit region. Since the DNA 5′ and 3′ ends of the nucleosome naturally exchange between wrapped and unwrapped states on short-time scales due to thermal fluctuations [38, 87, 88], the entry/exit region is already the most accessible in the unmodified state, making its relative accessibility rather insensitive to the effect of charge-altering site modifications. Yet these previously studied acetylation sites have measurable biological impact, consistent with the experimental observation [68] that changes in the DNA accessibility as small as 1.5-fold can have biological implications.

H3K64^Ac^ and H2BK57^Ac^ are discussed in the Additional file 1.

## Conclusion

Future progress in epigenetics will likely depend on our ability to understand the mechanisms through which the cell can selectively and reversibly destabilize the highly stable histone–DNA complex, thereby making nucleosomal DNA more accessible to cellular machinery responsible for vital functions such as transcription. Charge-altering post-translational modifications (PTM) in the globular histone core are one such mechanism that is beginning to emerge. However, so far progress was limited due to the vast amount of potential PTMs, the difficulties in exploring their effects experimentally, and the lack of a unifying framework that could rationalize the entire picture. Nucleosome assembly is another key biological process where a complete mechanistic understanding of the nucleosome stability control, currently lacking, is important. Charge-altering post-translational modifications of the globular histone core of the nucleosome, most notably acetylation of lysines, are good candidates for the nucleosome “control knobs”: These can disrupt favorable electrostatic interactions in this highly charged system. The altered electrostatics can affect the histone–histone and histone–DNA affinity and, therefore, change the stability of the nucleosome and accessibility of its DNA to cellular machinery.

Our main result is a physics-based, parameter-free framework that offers a quantitative, causal connection between any charge-altering PTM in the globular histone core, most notably lysine acetylation, and the nucleosome stability. Through the atomistic structure of the nucleosome and its key partially assembled states, the framework provides a connection between any charge-altering PTM to accessibility of its DNA in several regions, including the all-important entry/exit. Our main overall conclusion is an unexpected richness of the PTM landscape of the globular histone core. Based on the growing body of experimental evidence and the predictions presented here, it is our anticipation that further exploration of PTMs in the relatively unexplored globular core could provide as much biological insight as the well studied PTMs on histone tails with respect to controlling accessibility of the nucleosomal DNA.

Specifically, the effect of charge-altering PTMs on DNA accessibility can vary dramatically, from virtually none to a strong, region-dependent increase in accessibility of the nucleosomal DNA; in some cases, e.g., H4K44, H2AK75, and H2BK57, the effect is significantly stronger than that of the extensively studied acetylation sites such H3K56, H3K115, or H3K122. Most, but not all charge-altering PTMs are predicted to result in enough accessibility increase to enhance transcription. Furthermore, the model predicts that some PTM sites far from the DNA, e.g., H4K91, can still have a significant effect on its accessibility, while PTM sites four times closer to the DNA, e.g., H3K115, have only a minor effect. Completely counterintuitively, a small minority of possible acetylations, notably H4K77 and H2BK125, are predicted to decrease in DNA accessibility, suggestive of the corresponding repressed chromatin phenotype. While for the majority of the charge-altering PTMs, the effect on DNA accessibility is additive (noncooperative), there are exceptions, e.g., H4K79 and H3K122, where the combined effect is amplified. The amplification is a direct consequence of the nucleosome–DNA complex having more than two structural states.

With the PTM-accessibility link established, we show how our framework can aid reasoning about possible influence of a given PTM on biological processes that are known to depend directly on the DNA accessibility. In the case of transcription, where even very small and sequence non-specific changes in the accessibility can have measurable effect, we are able to utilize the full quantitative power of the framework to rationalize in vivo experiments and make a variety of testable predictions. We have introduced the concept of nucleosomal DNA accessibility PTMprint based on the DNA region(s) affected by each PTM and the corresponding accessibility change: PTMs with the same PTMprint may be expected to have similar biological impact within a similar genomic context.

Complex and costly experiments have so far investigated, with respect to influence on the nucleosome stability and DNA accessibility, only a handful of PTMs out of hundreds that potentially exist in the histone core. This very limited data set cannot yield statistically significant conclusions or lead to generalizations about the magnitude, sign, possible cooperativity, and underlying physical reason behind PTM effects on the DNA accessibility. In contrast, within our framework we have systematically investigated almost all possible charge-altering PTMs, including a statistically significant number of pairs of PTMs. Predictions we have made include a number of explanations for the previously observed PTM effects, as well as several completely counterintuitive observations. A large number of general testable predictions about core histone PTMs are made. Robust explanations are provided. We have also made a number of testable predictions about specific potential PTMs that may be “interesting” to investigate further, based on their PTMprints. For example, the acetylation of H4K44 is predicted to lead to a wide range of strong biological effects. While a full characterization of the biological, in vivo impact of each potential PTM site cannot be expected from a theoretical work, the proposed framework can help rationalize existing experiments and form working hypothesis for future ones.

Despite the high complexity of the nucleosome system and its partially assembled intermediate structures, the proposed framework provides a quantitative agreement with experiment with respect to the effects of the known PTMs on thermodynamic stability of the DNA–histone complex. It is noteworthy that the agreement is achieved via “first principle” calculations, without any fit to existing data on nucleosome stability. What may be even more noteworthy for a purely theoretical framework is that its predictions with respect to a complex in vivo process—transcription—are in agreement with observed in vivo effects of known PTMs, even in counterintuitive cases. This agreement gives us confidence in the model and its predictions. While no theoretical description of complex biological phenomena can claim 100% accuracy, and ours is not an exception, it is likely that our approach is still well above the “Null model” level.

Further thorough validation of the framework will be necessary for wide acceptance. Our predictions can be tested at several levels: (1) Thermodynamic measurements of predicted ΔΔ*G* values due to specific PTMs—only a handful are available now. Of particular interest can be strong effects and counterintuitive predictions of the model, such as net stabilizing lysine acetylations or highly nonadditive effect of select PTM pairs. (2) Likewise, general predictions of the model regarding specific PTM effect on transcription can be tested in a variety of biological contexts. Again, strong and counterintuitive effects can be good starting points, e.g., effects of H4K44^Ac^ or the possibility of observing transcriptionally repressed chromatin states for a handful of charge-altering PTMs (such as H4K77^Ac^) predicted to decrease the DNA accessibility.

Future extensions of the model can address some of its current limitations. These include the lower accuracy predictions for some of the charge-altering PTMs, most notably phosphorylations, the unknown effect of histone sequence variation, including histone variants, and, even more broadly, the inability of the current model to predict the effect on PTMs where no charge change occurs, such as methylation. Also, predictions of our current model reflect general trends, rather than being gene-specific: For certain individual genes the connection between the DNA accessibility and transcription level may not be direct, e.g., due to transcription enhancement of regulatory proteins that may down-regulate transcription. Further refinement of our model is possible by considering additional distinct regions of the nucleosomal DNA as an extension to the two regions in the current model. A finer breakdown of the PTM effects by the DNA region can be important since biological consequences of altered DNA accessibility are likely to be *context specific.* To generate context specific predictions based on our framework, it could be combined with a thermodynamic model of transcription regulation [89], which would allow it to predict PTM-induced changes in DNA accessibility relative to location of key regulatory elements such as transcription factor sites.

## Methods

### Multistate model of the nucleosome with varying degrees of DNA accessibility

Within our model, we consider the six, presumably most relevant biologically, and most extensively characterized experimentally, partially assembled or partially unwrapped structural states of the nucleosome [14, 22, 38, 59, 60, 67], Fig. 1, that the nucleosome–DNA system can occupy along its primary assembly and disassembly pathway. By comparing the calculated probabilities of the states post-PTM with the probabilities pre-PTM, our model estimates the relative change in probability of each state, which is then related to the change of DNA accessibility through the associated accessibility of the DNA in each structural state [62].

In addition to the intact (native) nucleosome, the partially assembled states are as follows. The tetrasome—a key structure that is known to occur on the nucleosome assembly pathway [22, 60]—composed of the (H3-H4)_2_ tetramer in complex with the nucleosomal DNA, of which 69 bp are accessible [62]. In addition, we also consider another important class of non-canonical nucleosome structures—the so-called partially unwrapped states of the nucleosomes [38, 67], which differ from the canonical nucleosome by the degree of “unpeeling” of its DNA.

In Fig. 1 the unwrapped state (U), the tetrasome state (T), the partially wrapped states (P1–P3), and the wrapped state (W) closely resemble the experimentally identified states VI, V, II, and I, respectively, from the notation of Andrews and Luger [14]. The generally accepted primary pathway to assembly [14, 67] is best represented in our notation as: U → T → P1–P3 → W.

Importantly, each of the conformational states provides accessibility to different regions of the nucleosomal DNA. We label the 147 bp of DNA as − 73 to 73 including 0 (the dyad position), Fig. 1. In the order of most to least amount of accessible base pairs, the states are as follows. State U consists of the DNA completely free of the histone proteins resulting in the DNA accessibility profile [− 73, 73]. State T consists of the (H3-H4)_2_ tetramer with 69 bp of the original DNA inaccessible; the removal of the H2A-H2B histones from the intact nucleosome exposes the DNA regions [− 73, − 34] and [34, 73]. The three partially wrapped states (P1, P2, and P3) are modeled such that 20 bp of DNA become accessible from either the 3′ (P1) or 5′ (P3) or 10 bp are accessible from both ends (P2), resulting in the DNA accessible regions of: [− 73, − 53] for P1, [− 73, − 63] and [63, 73] for P2, and [53, 73] for P3. And finally, state W is defined to have no DNA accessibility. We note that the partially wrapped states represent the DNA accessibility due to thermal fluctuations as seen in experiment [78, 88, 90].

### Structure preparation

The starting state for all of the nucleosome states considered here is the atomistic structure of the nucleosome core particle (PDB ID 1KX5) [12]. The protonation states of the ionizable residues were computed via the H++ server [91, 92] which employs the standard continuum electrostatics methodology for determining the pKs of amino acid residues [93]. We set the following parameters for estimating the protonation state of the nucleosome: 0.8 M of monovalent salt, *ɛ*_in_ = 12.5, *ɛ*_out_ = 80, and a pH value of 7.5. The value of *ɛ*_in_ = 12.5 was estimated as the volume averaged value between the DNA (*ɛ*_in_ = 15) and the core (*ɛ*_in_ = 4). The value of pH = 7.5 was used in the experiments that observed the unfolding at 0.8 M of monovalent salt [94] and serves as a good estimate of the pH inside the nucleus [95]. All other structural models described below “inherit” the same protonation state of the intact nucleosome.

The tetrasome is modeled by removing the H2A and H2B histones from the intact nucleosome, and by further removing the DNA in positions [− 73, − 34] and [34, 73]. It was shown recently [62] that the amount of protected DNA in the tetrasome is 69 ± 5 bp, and the (H3–H4)_2_ tetramer is structurally similar to that in the intact nucleosome, which justifies the choice. P1, P2, and P3 partially wrapped states are modeled from the intact nucleosome by removing 20 bp from the entry (P1) or exit (P3) ends of the nucleosomal DNA, or 10 bp from each end (P2).

### Modeling post-translational modifications

We have focused our study to potential PTM sites located within the globular histone core while excluding most of the sites on the C- and N-terminal tail regions [30]. Only the tail residues that are within 5 Å of the DNA were considered in our analysis, because these are close enough to the DNA “gyres” to be considered as “core,” at least in part. The key difference between the electrostatic effects of the core versus tail regions was discussed previously [25]. To mimic the change in charge of any of the PTM-modified residues, we alter a subset of the atomic partial charges within the modified residue, but do not change the number of atoms in the residue to avoid introducing steric clashes into the nucleosome structure that would likely result in unphysically high energy of the PTM states. The same “electrostatic only” approach is very successful in predicting pK and protonation state changes in proteins [91–93, 96–100]. By analogy, the smaller the steric changes introduced by a PTM, the better the accuracy of our approach.

We account for the two copies of each histone protein in the core by applying any PTM to both residues, e.g, acetylation of K56 on both H3 histones.

### Acetylation

The values of appropriate side-chain partial charges on LYS are reduced such that the total charge at neutral pH reduces from + 1 to 0, effectively mimicking de-protonation of the residue; the values of the atomic partial charges per residue type are given in the Additional file 1.

### Crotonylation, propionylation, butyrylation, and formylation

Crotonylation applies a minimal chemical modification to lysine that mimics the charge neutralization of the acetyl group applied during acetylation, but includes an allyl extension from the methyl group. Butyrylation is similar to crotonylation except the allyl extension is an ethyl group. Propionylation is less sterically intrusive than either crotonylation or butyrylation with just a single methyl extension from the acetyl group. And formylation is actually sterically preferred over acetylation as it replaces the methyl group with one hydrogen atom. The addition of at most two extra carbon atoms compared to the chemical modification involved in acetylation suggests that the predictions of our model, which assumes no significant disruption of the nucleosome due to steric interference of the PTM with the nearby groups, should also apply, at least semiquantitatively, for crotonylation, propionylation, butyrylation, and formylation. For crotonylation and butyrylation, we expect the results to be more qualitative since their modifications are two carbons larger than acetylation, which diminishes the accuracy of our model. With the above caveats, we use exactly the same protocol for these PTMs as described above for acetylation. Since the computational protocol is the same, all the computed values are the same, and thus the conclusions within our model made for the acetylations are expected to be similar for these PTM types.

### Phosphorylation

A similar approach was taken for the phosphorylation of the serine and threonine residues. Just as for the other PTMs, we did not explicitly model the phosphate group, but instead, we mimicked the effect of phosphorylation by altering the partial charges accordingly, see Additional file 1. However, in the case of phosphorylation, the approximation of minimal structural disruption caused by a PTM is less justified than for acetylation and other PTMs described above. Phosphorylation introduces a larger, sterically bulky and potentially more disruptive change to the histone core, which our model does not take into account. In addition, the phosphate group has multiple protonation states. For simplicity, we used the most probable (single) protonation state for all of the phosphorylations. The assumption is based on our pKa predictions for all SER, THR, and TYR in the nucleosome core, for which more than 90% are predicted in this protonation state. We used H++ webserver [91, 92] for these calculations. To the best of our knowledge, there is no experimental ΔΔ*G* data at physiological conditions available to us (The experimental ΔΔ*G* value is available for phosphorylated H3T118; however, this experiment was conducted under high salt conditions [47] which is not representative of in vivo conditions where our model was developed to be predictive). Given all of the above caveats and limitations, we treat our predictions for this PTM only as a general qualitative guide.

### Free energy calculation

We refer to the total free energy of the state without any modifications to the globular histone core charge as Δ*G*(native), and Δ*G*(PTM) refers to a state where a given PTM, *e.g.,* acetylation, was applied. We define ΔΔ*G* = Δ*G*(PTM) − Δ*G*(native), and compute this quantity for the acetylation of all the lysines in the globular histone core and for the phosphorylation of all the serines and threonines in the globular histone core. Note that we do not need to compute Δ*G*(native) or Δ*G*(PTM) separately, which would be subject to higher uncertainties than the difference between the two we seek.

To compute ΔΔ*G*, we make the approximation that the effect of the PTMs on the non-electrostatic components of Δ*G* is negligible compared to its effect on the electrostatic component, Δ*G*_electro_, such that ΔΔ*G* ≈ Δ*G*_electro_(PTM) − *ΔG*_electro_(native). This is a very reasonable assumption, especially for PTMs such as acetylation or crotonylation that result in minor steric modifications to the original structure. Essentially the same approach has been used successfully for decades to predict pKs of titratable groups in macromolecules based on their atomistic structures [91–93, 96–100]. Within the approach, local dynamics and flexibility are taken into account, albeit implicitly, through the dielectric response, which directly affects the estimated ΔΔ*G*.

A numerical solver for the nonlinear Poisson Boltzmann equation, APBS [101], was employed to compute changes in the nucleosome’s stability, ΔΔ*G*, due to changes in charge states of the histones. APBS was used with the following (standard) parameters: the internal dielectric of 4, the external dielectric of 80, and the monovalent salt concentration set to 145 mM with an ion radius of 2.0 Å. The boundary between the two dielectrics was set to be the molecular surface as determined by a probe radius of 1.4 Å. The nucleosome structure was centered in a box with each edge having a length of 360 Å. The box was uniformly divided into a grid containing 225^3^ grid points with a spacing of 1.607 Å between the points.

### The accessibility predictions are robust to key computational details

The main uncertainty of the continuum electrostatic calculations that our accessibility predictions are based upon comes from the use of a fixed, uniform dielectric constant for the solute (nucleosome + DNA) interior. All our predictions are based on the “standard” assumption of *ϵ*_in_ = 4; we have verified that our key verifiable predictions, ΔΔ*G* values in Table 1, remain within 0.4 kcal/mol if *ϵ*_in_ is increased by a factor of 2, from 4 to 8, which is a reasonable range for the internal dielectric. This variation, calculated for each ΔΔ*G* value in Table 1, is used as the error bar on the predicted ΔΔ*G* values.

### Change in accessibility $( {\text{P}}^{ *} / {\text{P)}}$ by DNA region

**Fig. 7:**
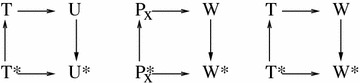
Thermodynamic cycles of the states used in the model

The formulation for accessibility for all six DNA regions in Fig. 1 is derived. The partition function, *Z*, without PTMs, for our six state (W, U, T, P1, P2, P3) model is:

$$\begin{aligned} Z & = e^{{ - \beta G_{U} }} + e^{{ - \beta G_{T} }} + e^{{ - \beta G_{P1} }} + e^{{ - \beta G_{P2} }} + e^{{ - \beta G_{P3} }} + e^{{ - \beta G_{W} }} \\ & = e^{{ - \beta G_{W} }} \left( {e^{{ - \beta \Delta G_{WU} }} + e^{{ - \beta \Delta G_{WT} }} + e^{{ - \beta \Delta G_{{WP_{1} }} }} + e^{{ - \beta \Delta G_{{WP_{2} }} }} + e^{{ - \beta \Delta G_{{WP_{3} }} }} + 1} \right) \\ & = ze^{{ - \beta G_{W} }} \\ \end{aligned}$$

where *G*_*X*_ is the free energy of state *X*, $\Delta G_{XY} = G_{Y} - G_{X}$ is the free energy difference between state *Y* and state *X* (where *Y* or *X* can be any of W, U, T, P P2, P3) as shown in the thermodynamic cycles, see Fig. 7, and $z = Z/e^{{ - \beta G_{W} }}$. Similarly, the partition function, with a PTM, is $Z^{*} = z^{*} e^{{ - \beta G_{{W^{*} }} }}$.

Absolute accessibility, the Boltzmann probability of states, without PTMs, where the DNA region *r* is accessible, is

$$P_{r} = \mathop \sum \limits_{{x = {\text{acc }}\,\,{\text{states}}}} e^{{ - \beta G_{x} }} /Z$$

where *G*_*x*_ represents the free energies of the *x* states where region *r* of the DNA is accessible. The relative change in absolute accessibility due to a PTM is,

$$P_{r}^{*} /P_{r} = \frac{{\mathop \sum \nolimits_{{x = {\text{acc }}\,\,{\text{states}}}} e^{{ - \beta G_{x}^{*} }} }}{{Z^{*} }}\frac{Z}{{\mathop \sum \nolimits_{{x = {\text{acc}}\,\,{\text{states}}}} e^{{ - \beta {\text{x}}}} }}$$

where *P*_r_^*^ is the Boltzmann probability of DNA region *r* being accessible, with a PTM, and ${\text{G}}$ are the free energies, with a PTM, for states *x* where region *r* of the DNA is accessible. The change in absolute accessibility for the entry/exit (e) and globally accessible (g) regions is as follows:

$$\begin{aligned} P_{e}^{*} /P_{e} & = \frac{{\left( {e^{{ - \beta T^{*} }} + e^{{ - \beta U^{*} }} + e^{{ - \beta P_{1}^{*} }} + e^{{ - \beta P_{2}^{*} }} + e^{{ - \beta P_{3}^{*} }} } \right)ze^{ - \beta W} }}{{\left( {e^{ - \beta T} + e^{ - \beta U} + e^{{ - \beta P_{1} }} + e^{{ - \beta P_{2} }} + e^{{ - \beta P_{3} }} } \right)z^{*} e^{{ - \beta W^{*} }} }} \\ & = e^{{ - \beta (\Delta G_{{W^{*} W}} + \Delta G_{{TT^{*} }} )}} \frac{z}{{z^{*} }}\frac{{1 + e^{{ - \beta (\Delta G_{{T^{*} T}} + \Delta G_{TU} + \Delta G_{{UU^{*} }} )}} + e^{{ - \beta (\Delta G_{{T^{*} T}} + \Delta G_{TW} - \Delta G_{PW} )}} \left( {e^{{ - \beta \Delta G_{{P_{1} P_{1}^{*} }} }} + e^{{ - \beta \Delta G_{{P_{2} P_{2}^{*} }} }} + e^{{ - \beta \Delta G_{{P_{3} P_{3}^{*} }} }} } \right)}}{{1 + e^{{ - \beta \Delta G_{TU} }} + 3e^{{ - \beta (\Delta G_{TW} - \Delta G_{PW} )}} }} \\ \end{aligned}$$

$$\begin{array}{*{20}c} {P_{g}^{*} /P_{g} } & = & {e^{{ - \beta (\Delta G_{{W^{*} W}} + \Delta G_{{TT^{*} }} )}} \frac{z}{{z^{*} }}\frac{{1 + e^{{ - \beta (\Delta G_{{T^{*} T}} + \Delta G_{TU} + \Delta G_{{UU^{*} }} )}} }}{{1 + e^{{ - \beta \Delta G_{TU} }} }}} \\ \end{array}$$

In the above formulation, the entry/exit region (e) is considered accessible in all but the Wrapped state, while the global region (g) is considered accessible in the Unwrapped and Tetrasome states shown in Fig. 1.

## Additional file

**Additional file 1.** Supplementary information to: modulation of nucleosomal DNA accessibility via charge altering post-translational modifications in histone core.

## Abbreviation

PTM: post-translational modification
