# Supplementary material for: Modulation of nucleosomal DNA accessibility via charge-altering post-translational modifications in histone core
Source: Epigenetics Chromatin. 2018 Mar 16;11:11. doi: 10.1186/s13072-018-0181-5 (PMC5856334; doi:10.1186/s13072-018-0181-5)
Supplement: Supplementary file 1 — Additional file 1. Supplementary information to: modulation of nucleosomal DNA accessibility via charge altering post-translational modifications in histone core. [file 13072_2018_181_MOESM1_ESM.docx]

Supporting Information

# *Modulation of Nucleosomal DNA Accessibility via Charge Altering Post-Translational Modifications Throughout the Histone Core.*

# Additional Results

## Accessibility and stability impact per core Acetylation PTM

The free energy change in nucleosome stability, $\Delta\Delta G$, due to the acetylation of each core lysine is shown in Table S1.

#### Lysine acetylation stability impact ($\boldsymbol{\Delta\Delta G}$)

Table S1. Destabilization estimates of the nucleosome due to the acetylation of specific residues in the globular core for all of the histones.

| Histone | Residue | $\Delta\Delta G$ (kcal/mol) |
| --- | --- | --- |
| H4 | LYS 91 | 7.4 |
| H4 | LYS 44 | 7.7 |
| H4 | LYS 79 | 1.7 |
| H4 | LYS 59 | 0.8 |
| H4 | LYS 16 | 0.4 |
| H4 | LYS 31 | 0.2 |
| H4 | LYS 20 | 0.1 |
| H4 | LYS 5 | -0.1 |
| H4 | LYS 12 | -0.6 |
| H4 | LYS 77 | -3.1 |
| H3 | LYS 37 | 2.7 |
| H3 | LYS 27 | 1.6 |
| H3 | LYS 56 | 2.3 |
| H3 | LYS 36 | 1.1 |
| H3 | LYS 122 | 0.3 |
| H3 | LYS 115 | 0.2 |
| H3 | LYS 64 | -0.1 |
| H3 | LYS 79 | -0.9 |
| H2B | LYS 28 | 13.0 |
| H2B | LYS 31 | 10.4 |
| H2B | LYS 27 | 5.8 |
| H2B | LYS 43 | 5.1 |
| H2B | LYS 57 | 4.8 |
| H2B | LYS 46 | 4.3 |
| H2B | LYS 24 | 3.5 |
| H2B | LYS 34 | 3.3 |
| H2B | LYS 23 | 2.7 |
| H2B | LYS 85 | 1.8 |
| H2B | LYS 108 | 1.2 |
| H2B | LYS 120 | 0.9 |
| H2B | LYS 116 | -0.1 |
| H2B | LYS 125 | -0.2 |
| H2A | LYS 13 | 8.8 |
| H2A | LYS 75 | 6.6 |
| H2A | LYS 36 | 2.0 |
| H2A | LYS 74 | 2.3 |
| H2A | LYS 119 | 1.5 |
| H2A | LYS 15 | 1.4 |
| H2A | LYS 125 | 1.4 |
| H2A | LYS 127 | 1.2 |
| H2A | LYS 118 | 1.6 |
| H2A | LYS 95 | 0.2 |
| H2A | LYS 129 | 0.4 |

## PTMprint: additional specific examples.

**H3K64^Ac^**. This particular residue in H3 histone, K64, has a peculiar predicted response when acetylated. For the global region, the relative increase in accessibility is low and unremarkable when compared to the other acetylation sites in H3. However, in the entry/exit region, H3K64 shows a relatively large increase in accessibility, see Figure 3. This suggests that while the overall impact of acetylating H3K64 could be relatively small, accessibility to certain DNA regions could still be meaningfully altered. In fact, recent work has shown that H3K64^Ac^ facilitates nucleosome eviction, alters nucleosome stability, and is enriched at the transcriptional start sites of active genes [1]. Curiously, enrichment of tri-methylated H3K64 is known to be in pericentric heterochromatin and other transcriptionally inactive genomic regions [2]. Furthermore, Daujat et al. hypothesized that tri-methylated H3K64 helps to 'secure' nucleosomes in a repressed state during development [2]. These opposing phenotypes from the different PTM types might suggest methylation as a “lock against acetylation” mechanism, activated when it is critical to keep certain regions of the genome inactive.

**H2BK57^Ac^**. By the PTMprint logic, yet uninvestigated H2BK57^Ac^ may be similar to H2BK46^Ac^. In yeast, mutating lysine to alanine (electrostatically mimicking acetylation) of H2BK49 (H2BK46 in *H. sapiens*) causes a significant growth defect when exposed to UV irradiation [3]. In human cells, H2BK46 is known to be acetylated and methylated. Additionally, preliminary studies involving the *H. sapiens* H2B variant suggest that levels of H2BK46^Ac^ might change in response to DNA damage induced by UV radiation [3].

## Accessibility and stability impact per core Phosphorylation PTM

Figure S1 details the change in relative accessibility, P*/P, for each phosphorylation site. The corresponding PTMprint classifications are provided in Table S2. The distribution of phosphorylation sites within the nucleosome, color-coded by the PTMprint classification, is shown in Figure S2. Quantitative estimates in the change in nucleosome stability, $\Delta\Delta G$, due to the phosphorylation of a core threonine, tyrosine or serine are provided in Table S3.

Figure S1. Nucleosomal DNA accessibility PTMprint for phosphorylated residues within the globular histone core. (a) Schematic of the different DNA regions. (b) Accessibility of the different nucleosomal DNA regions without a PTM. The very low probability of the global region accessibility corresponds to the simultaneously unwrapping of 78 bp of the DNA. Panel (c) shows change in accessibility, (*P^∗^/P*), upon phosphorylation, by regions Entry/Exit and Global. The horizontal dashed lines indicate the conservative threshold value used to define a functionally significant change in accessibility. The threshold value corresponds to the accessibility change of H4K31 for the entry/exit region, and to H4K79 for global accessibility. Note that the entry/exit nucleosomal DNA is accessible in multiple states (U, T, P1, P2, P3); the global region is only accessible in the T and U states, Figure 1 in the main text.

Table S2. PTMprint classification for all possible phosphorylations per core histone. PTMs predicted to have non-weak *stabilizing* effect on the nucleosome, and thus a *decrease* in the DNA accessibility, are marked by asterisk.

| Classification / Histone | H2A | H2B | H3 | H4 |
| --- | --- | --- | --- | --- |
| Change DNA accessibility in Entry/Exit | S122, S123 |  | S28, Y54 | Y51 |
| Change DNA accessibility Globally | T16, S18, S19, Y50, T59, T101 | T21, T32, S36, Y37, Y40, S64, S78, Y83, S87, T90, S91, T96, T115, Y121 | S87, Y99* | Y30, S47, T71, Y72, Y88, T96 |
| Change DNA accessibility in both regions | T76, T120, S125, S127 | Y42, T52, S55, S56, S60, T88 | Y41, T45, S57, T58, T118 | T80, Y98 |
| Weak effect on DNA accessibility | Y39, Y57, S113 | S112, T119, T122, S123 | T32, T80, S86, S96, T107 | T54, T73, T82 |

#### Distribution of phosphorylated residues throughout the core


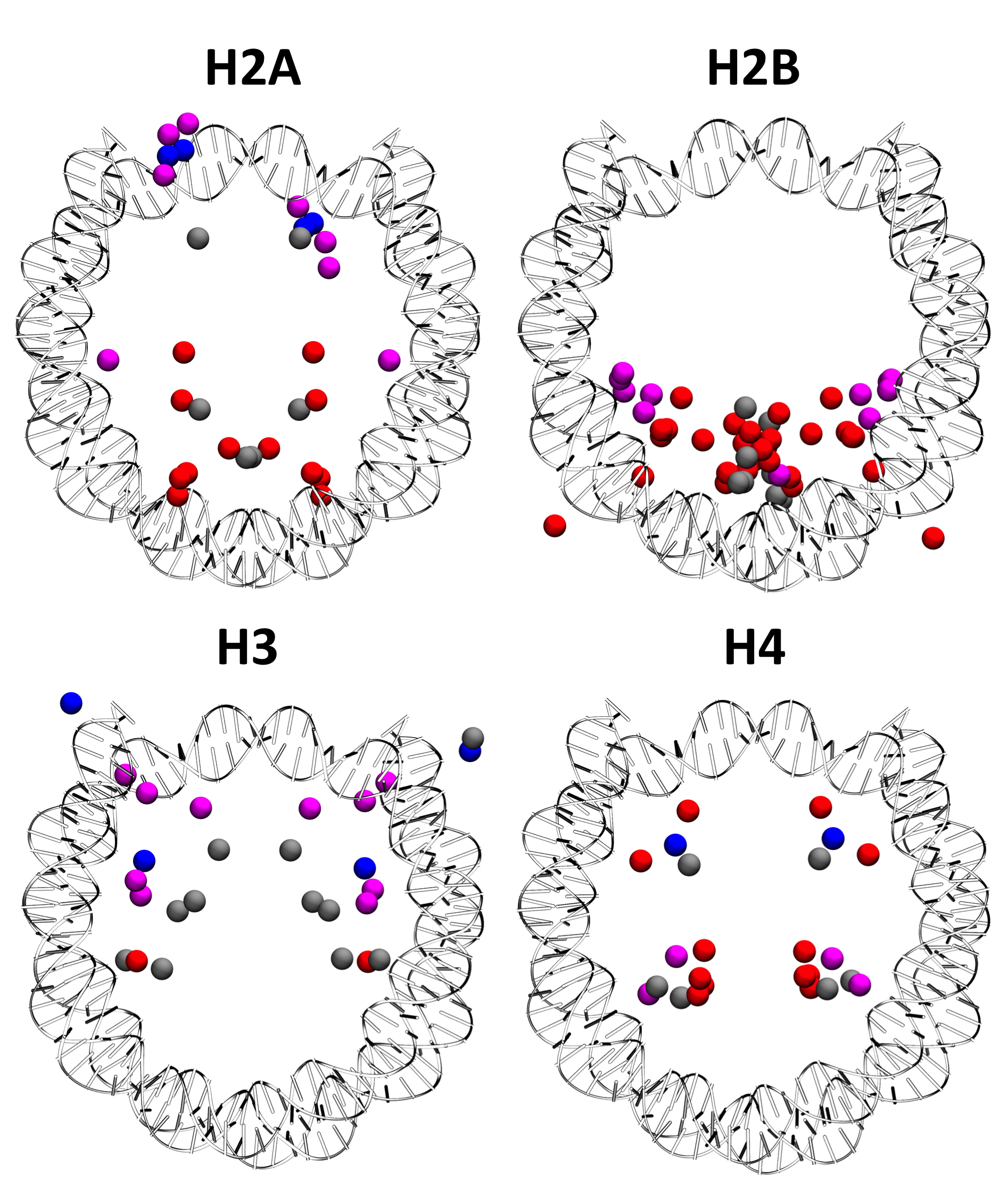


Figure S2. Spatial distribution of residues susceptible to phosphorylation throughout each histone. Each PTM for a given histone is color coded (Figure 4, main text) according to its DNA accessibility PTMprint: its predicted change on accessibility of different regions of the DNA, see table S2.

#### Serine, threonine, and tyrosine phosphorylation stability impact ($\boldsymbol{\Delta\Delta G}$)

Table S3. Destabilization estimates of the nucleosome due to the phosphorylation of specific residues in the globular core for all of the histones. A change in free energy exceeding 8.1 kcal/mol would result in a change in the protonation state of the phosphate group (see Methods below). Therefore we expect the upper limit of $\boldsymbol{\Delta\Delta G}$ to be 8.1 kcal/mol.

| Histone | Residue | $\Delta\Delta G$ (kcal/mol) |
| --- | --- | --- |
| H4 | TYR 98 | 8.1 |
| H4 | THR 80 | 8.1 |
| H4 | TYR 72 | 8.1 |
| H4 | THR 71 | 5.3 |
| H4 | THR 96 | 4.6 |
| H4 | THR 30 | 4.2 |
| H4 | SER 47 | 2.7 |
| H4 | TYR 88 | 1.8 |
| H4 | TYR 51 | 1.6 |
| H4 | THR 82 | 0.9 |
| H4 | THR 73 | 0.5 |
| H4 | THR 54 | -0.6 |
| H3 | THR 118 | 8.1 |
| H3 | THR 58 | 8.1 |
| H3 | SER 57 | 2.5 |
| H3 | SER 87 | 2.3 |
| H3 | THR 45 | 1.4 |
| H3 | SER 86 | 0.4 |
| H3 | SER 28 | 0.4 |
| H3 | TYR 41 | 0.4 |
| H3 | THR 107 | -0.7 |
| H3 | THR 32 | -1.1 |
| H3 | THR 80 | -1.1 |
| H3 | SER 96 | -1.3 |
| H3 | TYR 54 | -1.5 |
| H3 | TYR 99 | -5.4 |
| H2B | THR 88 | 8.1 |
| H2B | THR 32 | 8.1 |
| H2B | SER 87 | 8.1 |
| H2B | TYR 40 | 8.1 |
| H2B | SER 64 | 8.1 |
| H2B | THR 96 | 8.1 |
| H2B | SER 60 | 8.1 |
| H2B | SER 36 | 8.1 |
| H2B | SER 56 | 8.1 |
| H2B | TYR 42 | 8.1 |
| H2B | TYR 83 | 7.5 |
| H2B | SER 55 | 7.1 |
| H2B | THR 90 | 6.7 |
| H2B | TYR 121 | 6.0 |
| H2B | THR 52 | 5.6 |
| H2B | TYR 37 | 4.2 |
| H2B | THR 21 | 4.0 |
| H2B | THR 115 | 3.2 |
| H2B | SER 91 | 3.1 |
| H2B | SER 78 | 2.5 |
| H2B | THR 119 | 0.4 |
| H2B | THR 122 | 0.2 |
| H2B | SER 123 | -0.6 |
| H2B | SER 112 | -2.0 |
| H2A | THR 76 | 8.1 |
| H2A | THR 101 | 8.1 |
| H2A | SER 18 | 7.6 |
| H2A | SER 19 | 5.8 |
| H2A | THR 16 | 5.8 |
| H2A | SER 126 | 4.3 |
| H2A | SER 128 | 4.0 |
| H2A | THR 59 | 2.8 |
| H2A | THR 120 | 2.6 |
| H2A | TYR 50 | 1.9 |
| H2A | SER 122 | 1.2 |
| H2A | TYR 39 | 1.1 |
| H2A | SER 123 | 0.8 |
| H2A | TYR 57 | -1.3 |
| H2A | SER 113 | -2.4 |

#### PTMprint correlation with PTM distance to the DNA

We can gain insight into how the location of the charge altering modifications, such as acetylation, may be correlated with the ability of the PTM to modulate the accessibility of the different regions by considering the spatial proximity of the PTM relative to the nucleosomal DNA, Figure S3. Many potential acetylation sites across all histones that which modulate DNA accessibility strongly are in a close proximity to DNA (<12.5 Angstroms), while sites that are far away tend to have a weak effect. Therefore, while a general trend appears to be intuitive: an increase in the distance to DNA tends to lower the impact of the site (shifts into weak accessibility changes), there are many notable exceptions: H4K91 is far from the DNA, 19.5 Angstroms, and yet is predicted to globally increase DNA accessibility. Also, many sites in close proximity to the DNA are predicted to have a weak effect on DNA accessibility: H4K77, H4K20, H3K122, H3K115, H3K79, and H2B125. Further analysis (results not shown) reveals that both weak and strong PTM sites can be buried deep inside the protein core, or be close to the surface. Thus, "prediction" of acetylation effect on DNA accessibility based on intuitive proximity of a PTM to the DNA and histone surface is generally not possible.

Figure S3. Distribution of potential acetylation sites as a function of the distance to the nucleosomal DNA.

#### Correlation with known experimental observations

The model predictions for a multitude of PTMs correlate with a variety of known experimental observations, see Table S4.

Table S4. Experimentally characterized acetylations of the core histones and their observed phenotypes in-vivo. Two PTMs (in bold) are used here to set the thresholds of biological significance for the predicted PTM effect. Note that available experimental observation focus on disparate characteristics of response to PTMs, making it impossible to draw statistically significant conclusions regarding the correlation of a PTM with the biological outcome, with the possible exception of transcription enhancement as a direct effect of acetylation.

| **Residue** | **Physical Location** | **Classification** | **Predicted Entry/Exit and Global Accessibility Change** | **Experimental Observations** |
| --- | --- | --- | --- | --- |
| H3K56 | Entry/Exit | Entry/Exit and Global | 46 / 47 | DNA Breathing ^a^  Increase DNA Accessibility ^a, c^  DNA Repair ^a, b^  Increase Transcription ^a, b^  Replication ^b^ |
| H3K64 | Entry/Exit | Entry/Exit | 2.1 / 0.84 | Heterochromatin Formation ^a^  Increase Transcription ^b^  Nucleosome Destabilization ^c^ |
| H3K79 | Middle of core | Weak | 1.0 / 0.23 | Increase Transcription ^b^ |
| H3K115 | Dyad | Weak | 1.1 / 1.4 | Disassembly / Destabilization of the Nucleosome ^a, c^  Increase Transcription ^a^ |
| H3K122 | Dyad | Weak | 1.1 / 1.6 | Disassembly / Destabilization of the Nucleosome ^a, c^  Increase Transcription ^a, b^ |
| H4K31 | Entry/Exit | Entry/Exit | 1.4 / 1.5 | Reduced Viability ^a^ |
| H4K77 | Nearly opposite the dyad | Global Stabilizing | 1.0 / 0.0058 | Repressed chromatin ^e^ |
| H4K79 | Nearly opposite the dyad | Global | 1.0 / 19 | Increase DNA Accessibility ^a, c^  Loss of telomeric Silencing ^e^ |
| H4K91 | Center of core and deeply buried | Global | 1.1 / 2.6*${10}^{5}$ | Replication ^b^  Histone-Histone Destabilization ^b^ |

Relevant Reviews: ^a^[4], ^b^[5], ^c^[6], ^e^[7].

## Additivity of multiple PTMs

Here we provide a general thermodynamics-based explanation for why most of the PTMs are additive, and why the select few are not.

It is now well-recognized in the field that the nucleosome exists as multiple, interconverting structural states, including the ones used in our model, which means that cooperative behavior of some PTMs can be expected. However, most of the randomly chosen pairwise PTM cases shown in Figure 4 of the main text are additive (non-cooperative). This is because, for most acetylations, the multi-state model mimics a two-state one: the Boltzmann probabilities of the other states are relatively small and can be ignored. Specifically, the free energy *G* for one of the accessible nucleosome states in the model is significantly more favorable than the other accessible states, and the free energy *G* for one of inaccessible states is much more favorable than that of all the other inaccessible states. The presence of only two dominant states thus approximates a two-state system, which always behaves additively, and thus explains the “majority additive” finding. For the two exceptions noted in the main text (H4K79 and H4K44 pair and H4K79 and H3K122 pair), the free energy for the tetrasome accessible state for H4K79^Ac^ is not as dominant compared to the other PTMs considered here, and the two-state model is no longer a good approximation.

A quantitative analysis is presented below.

Figure S4. Thermodynamic cycles for illustrative 2- and 3-state systems. W (wrapped) and P (partially wrapped) are DNA inaccessible states and U (unwrapped) is a DNA accessible state.

Consider for example the thermodynamic cycle for a two state system with the DNA inaccessible state $W$ and the DNA accessible state $U$ shown in Figure S4 (left). The change in binding affinity due to PTMs 1 and 2 is

$$\begin{matrix} \Delta\Delta G^{1+2} & = & \Delta G_{W^{1+2}U^{1+2}}-\Delta G_{WU} \\ & = & G_{U^{1+2}}-G_{W^{1+2}}-G_{U}+G_{W} \\ & = & G_{U^{1+2}}-G_{U^{1}}-G_{W^{1+2}}+G_{W^{1}}+G_{U^{1}}-G_{U}-G_{W^{1}}+G_{W} \\ & = & \Delta G_{UU^{1}}+\Delta G_{U^{1}U^{1+2}}-\Delta G_{WW^{1}}-\Delta G_{W^{1}W^{1+2}} \\ & = & \Delta\Delta G^{1}+\Delta G_{U^{1}U^{1+2}}-\Delta G_{W^{1}W^{1+2}} \\ & = & \Delta\Delta G^{1}+\Delta G_{UU^{1+2}}-\Delta G_{UU^{1}}-\Delta G_{WW^{1+2}}+\Delta G_{WW^{1}} \end{matrix}$$

Within linear response electrostatics, the potentials, and hence the change in electrostatic energy of the system due to charge modifications at two separate sites are additive: $\Delta G_{WW^{1}}+\Delta G_{WW^{2}}=\Delta G_{WW^{1+2}}$ and $\Delta G_{UU^{1}}+\Delta G_{UU^{2}}=\Delta G_{UU^{1+2}}$. Then

$$\begin{matrix} \Delta\Delta G^{1+2} & = & \Delta\Delta G^{1}+\Delta G_{UU^{2}}-\Delta G_{WW^{2}} \\ & = & \Delta\Delta G^{1}+\Delta\Delta G^{2} \end{matrix}$$

which means strict additivity of the two PTMs. (We find that the deviations from the non-additivity due to the use of the non-linear PB are small).

For the 2-state system described above, the change in free energy between inaccessible and accessible states can be expressed as the difference in free energy between two states. For example, $\Delta G_{WU}=\beta^{-1}\ln\left[ \frac{e^{-\beta W}}{e^{-\beta U}} \right]=G_{U}-G_{W}$, which allowed us to simplify the formulation for change in binding affinity due to PTMs 1 and 2 as $\Delta\Delta G^{1+2}=\Delta\Delta G^{1}+\Delta\Delta G^{2}$. However, for the 3-state system shown in Figure S4 (right) with two DNA inaccessible states $W$ and $P$ and one DNA accessible state $U$.

$$\Delta G_{(WP)(W^{1}P^{1})}=\frac{1}{\beta}\ln\left[ \frac{e^{-\beta W}+e^{-\beta P}}{e^{-\beta W^{1}}+e^{-\beta P^{1}}} \right]\neq G_{W^{1}}+G_{P^{1}}-G_{W}-G_{P}$$

Therefore, even when $\Delta G_{WW^{1}}+\Delta G_{WW^{2}}=\Delta G_{WW^{1+2}}$ and $\Delta G_{PP^{1}}+\Delta G_{PP^{2}}=\Delta G_{PP^{1+2}}$

$$\Delta G_{(WP)(W^{1+2}P^{1+2})}\neq\Delta G_{(WP)(W^{1}P^{1})}+\Delta G_{(WP)(W^{2}P^{2})}$$

The above non-linear relationship from combining the Boltzmann distribution of multiple DNA inaccessible states, results in the non-additivity of PTMs.

For the specific 6-state system considered in this study (Figure 1 of the main text), Eq. (1) in the Methods section below, can be restated as:

$$\begin{matrix} \Delta\Delta G & = & -\beta^{-1}\ln\left[ e^{-\beta\Delta G_{WW^{*}}}+e^{-\beta\Delta G_{WP}}\left( e^{-\beta\Delta G_{P_{1}P_{1}^{*}}}+e^{-\beta\Delta G_{P_{2}P_{2}^{*}}}+e^{-\beta\Delta G_{P_{3}P_{3}^{*}}} \right) \right] \\ & & {+\beta}^{-1}\ln\left[ e^{-\beta\Delta G_{TT^{*}}}+e^{-\beta\left( \Delta G_{TU}+\Delta G_{UU^{*}} \right)} \right] \\ & & {+\beta}^{-1}\ln\left[ 1+3e^{-\beta\Delta G_{WP}} \right]-\beta^{-1}\ln\left[ 1+3e^{-\beta\Delta G_{TU}} \right] \end{matrix}$$

Let $\Delta\Delta G^{a+b}$ be the change in binding free energy when two sites $a$ and $b$, with binding free energy $\Delta\Delta G^{a}$ and $\Delta\Delta G^{b}$ for each of the two sites separately. In the above equation, $\Delta\Delta G^{a+b}\neq\Delta\Delta G^{a}+\Delta\Delta G^{b}$ even if $\Delta G^{a+b}=\Delta G^{a}+\Delta G^{b}$ for all $\Delta G$. However, if $T>>U$ or $U>>T$, and $W>>P$ or $P>>W$, then we have a two state system – one DNA accessible state and one DNA inaccessible state. Then, in the above equation, $\Delta G_{TU}$ and $\Delta G_{WP}$ are each either very large or very small, and the equation simplifies to a simple sum of $\Delta G$s without the exponentials. In such a system $\Delta\Delta G$ is additive. This is the case for most of the PTMs studied, and therefore in most cases the free energy change due to PTMs is additive. Otherwise, the degree of non-additivity depends on the difference between $\Delta G_{TT^{*}}$ and $\Delta G_{UU^{*}}+\Delta G_{TU}$ (DNA accessible states), and between $\Delta G_{WW^{*}}$ and $\Delta G_{WP^{*}}+\Delta G_{P_{x}P_{x}^{*}}$ (DNA inaccessible states).

# Methodological Details

## Modeling of charge-altering post translational modifications (PTM)

Our model mimics PTMs by changing the charge distribution of the modified sites. The changes in charge distributions due to acetylation and phosphorylation are provided in Tables S5-S8.

We note that a phosphorylated threonine, tyrosine and serine groups might either be in singly or doubly protonated state. These singly protonated groups have a net charge of -1e, as shown in tables S6-S8. We have assumed a singly protonated state for all of our computations. Adding a second proton would reduce the change in accessibility due to a smaller difference in net charge (-1e) compared to the charge of the unmodified group. Moreover, adding a second proton would entail an energetic cost of approximately 8.1 kcal/mol as estimated below. Therefore, if the change in free energy ($\Delta\Delta G$) were to exceed 8.1 kcal, the group would become doubly protonated thus limiting the change in free energy to 8.1 kcal/mol. Accordingly, the change in accessibility ($P^{*}/P$) has also been capped at an equivalent value of $\exp\left( \Delta\Delta G \right)= 3.39*{10}^{-3}$. The energetic cost of adding a proton to the phosphate group is based on the pKa of the transition from a singly protonated to a doubly protonated state, which is known to be approximately 1.5. The equivalent free energy difference at the physiological pH of 7.5 is equal to 2.3 kT * (pKa – pH) or 8.1 kcal/mol.

Table S5. The atom centered point charge conversion table for mimicking an acetylated lysine.

| Atom Type | Original Charge | Acetylated Charge |
| --- | --- | --- |
| N | -0.348 | -0.348 |
| H | 0.274 | 0.274 |
| CA | -0.240 | -0.240 |
| HA | 0.143 | 0.143 |
| CB | -0.009 | -0.009 |
| 2HB | 0.036 | 0.036 |
| 3HB | 0.036 | 0.036 |
| CD | -0.048 | 0.000 |
| 2HD | 0.062 | 0.000 |
| 3HD | 0.062 | 0.000 |
| CE | -0.014 | 0.000 |
| 2HE | 0.114 | 0.000 |
| 3HE | 0.114 | 0.000 |
| CG | 0.018 | 0.018 |
| 2HG | 0.010 | 0.010 |
| 3HG | 0.010 | 0.010 |
| NZ | -0.385 | -0.075 |
| 1HZ | 0.340 | 0.000 |
| 2HZ | 0.340 | 0.000 |
| 3HZ | 0.340 | 0.000 |
| C | 0.734 | 0.734 |
| O | -0.589 | -0.589 |

Table S6. The atom centered point charge conversion table for mimicking a phosphorylated serine.

| Atom Type | Original Charge | Phosphorylated Charge |
| --- | --- | --- |
| N | -0.4157 | -0.5163 |
| H | 0.2719 | 0.2936 |
| CA | -0.0249 | -0.0217 |
| HA | 0.0843 | 0.1229 |
| CB | 0.2117 | 0.0250 |
| 2HB | 0.0352 | 0.1052 |
| 3HB | 0.0352 | 0.1052 |
| OG | -0.6546 | -1.1120 |
| HG | 0.4275 | 0.0000 |
| C | 0.5973 | 0.5366 |
| O | -0.5679 | -0.5819 |

Table S7. The atom centered point charge conversion table for mimicking a phosphorylated threonine.

| Atom Type | Original Charge | Phosphorylated Charge |
| --- | --- | --- |
| N | -0.4157 | -0.5163 |
| H | 0.2719 | 0.2936 |
| CA | -0.0389 | 0.0439 |
| HA | 0.1007 | 0.1045 |
| CB | 0.3654 | 0.3527 |
| HB | 0.0043 | 0.0375 |
| CG2 | -0.2438 | -0.2875 |
| 1HG2 | 0.0642 | 0.0543 |
| 2HG2 | 0.0642 | 0.0543 |
| 3HG2 | 0.0642 | 0.0543 |
| OG1 | -0.6761 | -1.458 |
| 1HG | 0.4102 | 0.0000 |
| C | 0.5973 | 0.5366 |
| O | -0.5679 | -0.5819 |

Table S8. The atom centered point charge conversion table for mimicking a phosphorylated tyrosine.

| Atom Type | Original Charge | Phosphorylated Charge |
| --- | --- | --- |
| N | -0.4157 | -0.5163 |
| H | 0.2719 | 0.2936 |
| CA | -0.0014 | 0.2755 |
| HA | 0.0876 | 0.0082 |
| CB | -0.0152 | -0.3541 |
| 2HB | 0.0295 | 0.1103 |
| 3HB | 0.0295 | 0.1103 |
| CG | -0.0011 | 0.1197 |
| CD1 | -0.1906 | -0.1989 |
| HD1 | 0.1699 | 0.1371 |
| CE1 | -0.2341 | -0.2849 |
| HE1 | 0.1656 | 0.1772 |
| OH | -0.5579 | -1.1157 |
| CZ | 0.3226 | 0.4526 |
| HH | 0.3992 | 0.0000 |
| CE2 | -0.2341 | -0.2849 |
| HE2 | 0.1656 | 0.1772 |
| CD2 | -0.1906 | -0.1989 |
| HD2 | 0.1699 | 0.1371 |
| C | 0.5973 | 0.5366 |
| O | -0.5679 | -0.5819 |

## Change in binding affinity to DNA due to a PTM ($\boldsymbol{K}^{\boldsymbol{*}}\boldsymbol{/K}$)

In the main text we represented the effect of PTMs as the ratio of DNA accessibility (Boltzmann probability of accessible states) with and without the PTM, *P*/P*. Alternatively, the effect of PTMs could be represented as the ratio of equilibrium constant with and without the PTM, $K$**/K*. The equilibrium constant or binding affinity itself is the ratio of Boltzmann probabilities of the accessible and inaccessible states, and $\text{ln}\frac{K^{*}}{K}= \Delta\Delta G^{*}$, the change in free energy of binding due to the PTM. Since most experimental results are reported as the change free energy of binding, we use $\Delta\Delta G^{*}$ when comparing our results to experimental data, although accessibility (*P*) is arguably more intuitive and robust, and also produces a simpler formulation. For most cases the computed values for *change* in accessibility due to PTMs, *P*/P*, the quantity that we are ultimately interested in here, is approximately the same as *K*/K*. The formulation for *K*/K* is derived below.

We approximate the dynamical nature of the nucleosome via six separate states that represent known physical states of the nucleosome, see figure 1 in the main text. Two of the six states would provide transcriptional regulators access to the DNA, while the remaining four states model the DNA in a tightly wrapped geometry around the histone core and thus inhibit transcription of any occluded genes. We refer to this as the globally accessible region. The four states where the DNA remains mostly inaccessible, the globally accessible region, are the wrapped (W) and three partially wrapped ($P_{1}$, $P_{2}$ and $P_{3}$) nucleosomes. $P_{1}$ and $P_{3}$ represent partially wrapped nucleosomes with 20 bp unwrapped at either the 5’ or 3’ ends of the DNA, and $P_{2}$ represents the partially wrapped nucleosome with 10 bp unwrapped at both the 5’ or 3’ ends of the DNA. The binding affinity for the globally accessible DNA region is

$$\begin{matrix} K & = & \frac{e^{-\beta T}+e^{-\beta U}}{e^{-\beta W}+e^{-\beta P_{1}}+e^{-\beta P_{2}}+e^{-\beta P_{3}}} \\ & = & e^{-\beta(T-W)}\frac{1+e^{-\beta(U-T)}}{1+e^{-\beta(P_{1}-W)}+e^{-\beta(P_{2}-W)}+e^{-\beta(P_{3}-W)}} \\ & = & e^{-\beta\Delta G_{WT}}\frac{1+e^{-\beta\Delta G_{TU}}}{1+e^{-\beta\Delta G_{WP_{1}}}+e^{-\beta\Delta G_{WP_{2}}}+e^{-\beta\Delta G_{WP_{3}}}} \\ & = & e^{-\beta\Delta G_{WT}}\frac{1+e^{-\beta\Delta G_{TU}}}{1+3e^{-\beta\Delta G_{WP}}} \end{matrix}$$

where $W,P_{1},P_{2},P_{3},U,T$ are the free energies of the wrapped, partially wrapped, unwrapped and tetrasome states, and $\Delta G_{xy}=y-x$ is the change in free energy when going from state x to y. We also assume that $\Delta G_{WP_{1}}=\Delta G_{WP_{2}}=\Delta G_{WP_{3}}=\Delta G_{WP}$

Figure S5. Thermodynamic cycles within the model.

Similarly, $K^{*}$ the binding affinity when the core contains post translational modifications (^*^) can be calculated by applying the thermodynamic cycle shown in figure S5:

$$\begin{matrix} K^{*} & = & e^{-\beta\Delta G_{W^{*}T^{*}}}\frac{1+e^{-\beta\Delta G_{T^{*}U^{*}}}}{1+e^{-\beta\Delta G_{W^{*}P_{1}^{*}}}+e^{-\beta\Delta G_{W^{*}P_{2}^{*}}}+e^{-\beta\Delta G_{W^{*}P_{3}^{*}}}} \\ & = & \frac{e^{-\beta(\Delta G_{W^{*}W}+\Delta G_{WT}+\Delta G_{TT^{*}})}\left[ 1+e^{-\beta(\Delta G_{T^{*}T}+\Delta G_{TU}+\Delta G_{UU^{*}})} \right]}{1+e^{-\beta(\Delta G_{W^{*}W}+\Delta G_{WP_{1}}+\Delta G_{P_{1}P_{1}^{*}})}+e^{-\beta(\Delta G_{W^{*}W}+\Delta G_{WP_{2}}+\Delta G_{P_{2}P_{2}^{*}})}+e^{-\beta(\Delta G_{W^{*}W}+\Delta G_{WP_{3}}+\Delta G_{P_{3}P_{3}^{*}})}} \\ & = & \frac{e^{-\beta(\Delta G_{W^{*}W}+\Delta G_{WT}+\Delta G_{TT^{*}})}\left[ 1+e^{-\beta(\Delta G_{T^{*}T}+\Delta G_{TU}+\Delta G_{UU^{*}})} \right]}{1+e^{-\beta(\Delta G_{W^{*}W}+\Delta G_{WP})}\left( e^{-\beta\Delta G_{P_{1}P_{1}^{*}}}+e^{-\beta\Delta G_{P_{2}P_{2}^{*}}}+e^{-\beta\Delta G_{P_{3}P_{3}^{*}}} \right)} \end{matrix}$$

The change in affinity due to the post translational modification is defined as the ratio $K^{*}/K$

$$\begin{matrix} K^{*}/K & = & \frac{e^{-\beta(\Delta G_{W^{*}W}+\Delta G_{TT^{*}})}\left[ 1+e^{-\beta(\Delta G_{T^{*}T}+\Delta G_{TU}+\Delta G_{UU^{*}})} \right]\left[ 1+3e^{-\beta\Delta G_{WP}} \right]}{\left[ 1+e^{-\beta(\Delta G_{W^{*}W}+\Delta G_{WP})}\left( e^{-\beta\Delta G_{P_{1}P_{1}^{*}}}+e^{-\beta\Delta G_{P_{2}P_{2}^{*}}}+e^{-\beta\Delta G_{P_{3}P_{3}^{*}}} \right) \right]\left[ 1+e^{-\beta\Delta G_{TU}} \right]} \end{matrix} (1)$$

Experimentally determined values are used in the above formula for $\Delta G_{PW}$ and $\Delta G_{TU}$. The estimate of $\Delta G_{P_{1}W}=\Delta G_{P_{2}W}=\Delta G_{P_{3}W}=-3.1$ kcal/mol for a 20 bp long fragments was obtained via a linear regression over five available experimental measurements [8] for fragment lengths between 5 and 40 bp, as summarized in [9]. The estimate of $\Delta G_{TU}$ is from table 6.3 and 6.1 of Rippe, Mazurkiewicz and Kepper [10] such that $\Delta G_{TU}$ = +12.6 kcal/mol (tetrasome $\to$ disome) + 6.5 kcal/mol (disome $\to$ 2(H3-H4) + DNA) - 6.9 kcal/mol (2(H3-H4) $\to$ (H3-H4)_2_) = +12.2 kcal/mol. Values for $\Delta G_{X^{*}X}$ and $\Delta G_{XX^{*}}$ are determined computationally, as described in the main text.

## Estimating uncertainty in experimental *𝜟𝜟𝑮* for H3 LYS 56 acetylation

The experimental $\Delta\Delta G$ value for H3K56 acetylation was obtained from Table 1 of Andrews et. al. [11]. However, the table does not contain the uncertainty in the above experimental value. The table does however contain the experimental equilibrium constants for transitions between intermediate states, along with the uncertainty in each of those equilibrium constants, which we use here to estimate the uncertainties in the reported *𝜟𝜟𝑮.*  Specifically, Andrews et. al. identified seven states with seven transitions between these states, with the equilibrium constants labeled $K_{i}$. The table contains these equilibrium constants for three forms of the nucleosome: the 5S nucleosome ($K_{i}^{5S}$), the 601 nucleosome ($K_{i}^{601}$), and the 601 nucleosome with acetylated H3K56 ($K_{i}^{ac}$). The change in free energy due to acetylation is calculated as

$$\Delta\Delta G=\sum_{i=1}^{7} kT[\ln(K_{i}^{601}/K_{i}^{5S})+\ln(K_{i}^{ac}/K_{i}^{601})]$$

The propagation of error for the$kT \ln(K_{a}/K_{b})$ terms in the above summation are calculated as

$$err=kT\sqrt{(}(err_{b}/K_{b})^{2}+(err_{a}/K_{a})^{2}))$$

where $err_{a}$ and $err_{b}$ are the uncertainty in $K_{a}$ and $K_{b}$. The total error in the sum in $\Delta\Delta G$ is the square-root of the sum of squares of each of the terms in the summation.

# REFERENCES

1. Di Cerbo V, Mohn F, Ryan DP, Montellier E, Kacem S, Tropberger P, et al. Acetylation of histone H3 at lysine 64 regulates nucleosome dynamics and facilitates transcription. eLife. 2014;3:e01632–e01632.

2. Daujat S, Weiss T, Mohn F, Lange UC, Ziegler-Birling C, Zeissler U, et al. H3K64 trimethylation marks heterochromatin and is dynamically remodeled during developmental reprogramming. Nat Struct Mol Biol. 2009;16:777–81.

3. Kyriss MNM, Jin Y, Gallegos IJ, Sanford JA, Wyrick JJ. Novel Functional Residues in the Core Domain of Histone H2B Regulate Yeast Gene Expression and Silencing and Affect the Response to DNA Damage. Mol Cell Biol. 2010;30:3503–18.

4. Tropberger P, Schneider R. Scratching the (lateral) surface of chromatin regulation by histone modifications. Nat Struct Mol Biol. 2013;20:657–61.

5. Tessarz P, Kouzarides T. Histone core modifications regulating nucleosome structure and dynamics. Nat Rev Mol Cell Biol. 2014;15:703–8.

6. Bowman GD, Poirier MG. Post-Translational Modifications of Histones That Influence Nucleosome Dynamics. Chem Rev. 2014. doi:10.1021/cr500350x.

7. Hyland EM, Cosgrove MS, Molina H, Wang D, Pandey A, Cottee RJ, et al. Insights into the Role of Histone H3 and Histone H4 Core Modifiable Residues in Saccharomyces cerevisiae. Mol Cell Biol. 2005;25:10060–70.

8. Polach KJ, Widom J. Mechanism of Protein Access to Specific DNA Sequences in Chromatin: A Dynamic Equilibrium Model for Gene Regulation. J Mol Biol. 1995;254:130–49.

9. Garcia HG, Grayson P, Han L, Inamdar M, Kondev J, Nelson PC, et al. Biological consequences of tightly bent DNA: The other life of a macromolecular celebrity. Biopolymers. 2007;85:115–130.

10. Rippe K, Mazurkiewicz J, Kepper N. Interactions of histones with DNA: nucleosome assembly, stability, dynamics, and higher order structure. In: DNA Interactions with Polymers and Surfactants. John Wiley & Sons, Inc; 2008. p. 135–72.

11. Andrews AJ, Chen X, Zevin A, Stargell LA, Luger K. The Histone Chaperone Nap1 Promotes Nucleosome Assembly by Eliminating Nonnucleosomal Histone DNA Interactions. Mol Cell. 2010;37:834–42.
